# Network-Resolved and Frequency-Specific EEG–fMRI Representational Similarity Analysis of Visual Imagery

**DOI:** 10.3390/brainsci16101024

**Published:** 2026-09-25

**Authors:** Kaixin Huang, Lei Zhao, Fan Wang, Zhengshuang Li, Jingao Gao, Lixiang Ren, Yuhui Chen, Yunfa Fu, Tianwen Li

**Affiliations:** 1 Faculty of Science, Kunming University of Science and Technology, Kunming 650500, China; huangkaixin_hkx@163.com (K.H.); ynzhaolei@foxmail.com (L.Z.); 13578448661@163.com (Z.L.); 18183794848@163.com (J.G.); 2 Faculty of Information Engineering and Automation, Kunming University of Science and Technology, Kunming 650500, Chinafyf@ynu.edu.cn (Y.F.); 3 Department of Magnetic Resonance, The First People’s Hospital of Yunnan Province, Kunming 630100, China; renlixiang19830605@163.com (L.R.); yuhuichen1221@163.com (Y.C.)

**Keywords:** representational similarity analysis, visual imagery, electroencephalography, functional magnetic resonance imaging

## Abstract

**Background**: Visual imagery (VI) is a cognitive process that generates visual representations in the absence of external visual input; however, the spatiotemporal organization of its neural representations remains poorly understood. **Methods**: In this study, we combined electroencephalography (EEG) and functional magnetic resonance imaging (fMRI) with representational similarity analysis (RSA) and the Schaefer 2018 functional parcellation atlas to compare the correspondence between brain network representations and CORnet-S model representations during visual perception (VP) and VI. We further examined the cross-modal associations between EEG time–frequency representations and fMRI network representations during VI. **Results**: Significant model–brain representational correspondence was detected in the visual network during VP, and no significant correspondence was detected in any network during VI, yielding different descriptive significance profiles across the two tasks. Cross-modal EEG–fMRI RSA further revealed frequency- and time-dependent representational correspondence involving the frontoparietal, default mode, ventral attention, visual, and limbic networks. Significant cross-modal correspondence was observed across the alpha, beta, and gamma bands, with descriptive variation in the networks and temporal clusters involved in each frequency band. **Conclusions**: Collectively, these findings suggest that VI is not merely a reinstatement of VP but is associated with distributed representational patterns involving multiple functional networks. This study provides multimodal evidence for understanding the neural mechanisms underlying VI.

## 1. Introduction

Mental imagery plays a fundamental role in human cognition, and its study dates back to ancient Greek philosophy. Extensive empirical evidence has demonstrated that mental imagery supports higher-order cognitive processes, including memory encoding and retrieval, future event simulation, spatial navigation, and decision making [1]. Meanwhile, aberrant imagery processing has been implicated in the development and maintenance of various psychiatric disorders, such as post-traumatic stress disorder, depression, and anxiety disorders. Accordingly, imagery-based interventions have gradually emerged as important components of psychological treatment approaches [2,3,4]. Visual imagery (VI) refers to the cognitive process through which the brain internally generates visual representations in the absence of external visual stimulation [5,6,7]. As a major domain within mental imagery research, VI provides valuable insights into the neural mechanisms underlying endogenous visual processing and holds substantial potential for clinical translation. Therefore, investigating the neural mechanisms and regulatory pathways of VI is of dual significance for advancing theoretical understanding and facilitating practical applications.

Electroencephalography (EEG) provides millisecond-level temporal resolution and offers the advantages of portability and ease of operation, making it an effective tool for characterizing the dynamic neural processes underlying VI. Existing EEG studies have distinguished the neural mechanisms of VI from those of veridical visual perception (VP) across multiple dimensions, including cortical information flow, neural oscillations, event-related potentials (ERPs), and time–frequency dynamics. At the level of cortical information flow, Hamamé et al. (2012) used intracranial EEG recordings to demonstrate that broadband gamma oscillations (50–150 Hz) in the inferior temporal cortex were selectively enhanced during VI and visuospatial working memory tasks but showed no significant response to externally presented passive visual stimuli [8]. These findings provided evidence that VI does not simply represent a replay of externally driven sensory signals. Building on this work, subsequent studies have employed causal modeling approaches to characterize the directionality of information transmission between the two processes. Dentico et al. (2014), combining Granger causality analysis with dynamic causal modeling (DCM), demonstrated that VP is primarily supported by bottom-up cortical information flow, whereas VI relies on top-down feedback pathways, providing causal evidence for the hypothesis that the two processes involve distinct hierarchical patterns of information transmission [9]. More recently, Kim et al. (2024) combined a sensory substitution paradigm with effective connectivity analysis to identify two independent hierarchical processing modes underlying VI: top-down signal transmission is mediated by within-frequency neural coupling, whereas higher-level top-down modulation regulates lower-level neural activity through cross-frequency coupling mechanisms [10].

At the level of temporal and spectral characteristics of neural oscillations, accumulating evidence indicates that oscillatory activity across distinct frequency bands selectively encodes different types of visual imagery-related memories, attentional states, and subjective vividness. Kilmarx et al. (2024) compared VI during working memory retrieval and long-term memory retrieval and found significant differences in sustained alpha-band (8–13 Hz) oscillatory patterns between the two tasks, suggesting that the type of memory representation directly modulates the temporal dynamics of imagery generation [11]. Huang et al. (2023) investigated VI from the perspective of attentional regulation and reported that the amplitude of the vigilance-related N1 component during eyes-closed states was positively correlated with imagery vividness, demonstrating that internally oriented attentional focus is a critical factor regulating the subjective intensity of visual imagery experiences [12]. ERP-based studies have further provided evidence for temporal differences between VI and VP. Proverbio et al. (2023) showed that imagery-evoked ERP responses exhibited a delay of 200–400 ms relative to those elicited by veridical visual perception, with stronger visual category effects observed in the prefrontal cortex, supporting the notion that VI relies on top-down cognitive control and endogenous memory retrieval processes [13]. In addition, Alazrai et al. (2022) demonstrated, through time–frequency feature extraction approaches, that time–frequency representations across the theta, alpha, beta, and gamma bands exhibited high task specificity and could be effectively discriminated using deep learning models [14]. Together, these EEG studies have elucidated the millisecond-scale neural dynamics underlying VI. However, due to the limited spatial sampling of scalp electrodes, EEG cannot precisely localize fine-grained temporal neural signals to large-scale functional brain networks, thereby limiting the characterization of the macroscopic spatial architecture associated with VI.

Functional magnetic resonance imaging (fMRI), leveraging the excellent spatial localization capability of the blood-oxygen-level-dependent (BOLD) signal, has been widely used to identify the core brain regions and large-scale functional networks associated with visual imagery. Through large-scale meta-analyses and multivariate decoding approaches, accumulating evidence has established a dual-stream processing framework for visual imagery. Hawes et al. (2026) conducted an activation likelihood estimation meta-analysis of 46 fMRI and positron emission tomography (PET) experiments, revealing that spatial imagery selectively activated the bilateral dorsal visual pathway, whereas object imagery preferentially engaged the left ventral visual pathway, including the fusiform gyrus and lingual gyrus. In addition, both types of imagery shared overlapping activation patterns in the precuneus and medial frontal cortex, providing strong support for the dorsal–ventral dual-stream framework of visual imagery processing [15]. Using multivariate encoding and decoding models, Li et al. (2023) further demonstrated that the strength of visual orientation representations in the intraparietal sulcus during imagery tasks was significantly greater than that observed in the primary visual cortex, suggesting that parietal regions play a critical role in distinguishing internally generated imagery from externally driven visual experiences [16]. Alho et al. (2023) applied multivariate pattern analysis (MVPA) and showed that interference from external visual distractors on imagined contents occurred specifically in object-selective visual cortices, but not in higher-order frontoparietal control regions, refining our understanding of hierarchical visual representation from lower-level sensory regions to higher-order cognitive systems [17].

The role of the primary visual cortex (V1) in generating the subjective experience of visual imagery remains a central topic of debate and has been examined in a growing body of research. Clinical evidence suggests that visual imagery depends on distributed neural systems. Thorudottir et al. (2020) reported a case of imagery loss after posterior brain injury, underscoring the role of posterior visual regions and their broader networks [18]. Cabbai et al. (2024) conducted an fMRI study in individuals with aphantasia and found that although V1 could support the decoding of auditory categories during passive auditory tasks, it failed to support subjective visual imagery experiences. Moreover, the absence of precuneus decoding signals closely corresponded with impaired imagery ability, indicating that V1 activity alone is not sufficient for generating internally experienced visual imagery [19]. Sartin et al. (2026) further demonstrated under blindfolded conditions that tactile size representations encoded in V1 and the occipital pole could not be transferred to visual imagery tasks, whereas frontoparietal cortical regions supported cross-decoding between tactile exploration and VI tasks. These findings suggest that frontoparietal regions maintain abstract spatial representations that are independent of sensory modalities [20]. Time-series analyses of fMRI data have provided further support for a top-down reverse-processing framework. Hu et al. (2025) observed that during VI, decoding signals in V1 emerged significantly later than those in higher-order visual regions, including the lateral occipital complex and fusiform gyrus, whereas during visual perception, V1 activation preceded responses in higher-order visual cortices. These findings indicate that VI relies on reverse hierarchical processing to reconstruct visual representations across spatial and temporal levels [21]. At the level of large-scale network topology, Kvamme et al. (2025) employed large-scale structural–functional coupling network analyses and found that individual differences in imagery vividness were significantly associated with local network efficiency in the fusiform gyrus, clustering coefficient of occipital networks, and global network efficiency of the insula. These findings suggest that variability in visual imagery ability is rooted in whole-brain network topology [22]. Although fMRI provides a detailed characterization of the spatial organization of imagery-related networks, the inherently limited temporal resolution of BOLD signals at the scale of seconds restricts its ability to capture rapid fluctuations in neural oscillatory dynamics during imagery generation.

EEG and fMRI exhibit inherent complementarity in temporal and spatial resolution; however, simultaneous acquisition of these two modalities remains challenging due to practical limitations, including the high cost of MR-compatible hardware, severe gradient-induced artifacts, and stringent requirements for temporal synchronization. Consequently, studies involving concurrent EEG–fMRI acquisition remain relatively limited. Representational similarity analysis (RSA) provides a feasible alternative approach by enabling cross-modal mapping between temporal dynamics and spatial network representations without requiring simultaneous data acquisition. By quantifying the similarity structures of neural activity patterns across different experimental conditions, RSA effectively bridges neural representations across modalities while circumventing hardware constraints and signal interference associated with synchronous acquisition. Beyond facilitating cross-scale integration of multimodal neural data, RSA also provides a quantitative framework for linking computational models with brain activity, thereby offering a powerful approach for investigating hierarchical mechanisms of visual processing. In visual neuroscience, biologically inspired models such as CORnet have been widely used to simulate hierarchical processing along the primate ventral visual stream. Numerous studies employing model-based fMRI representational similarity analysis have demonstrated hierarchical correspondence between neural representations and model features, ranging from low-level feature-selective visual regions to higher-order semantic regions. However, how hierarchical representations underlying VI are coupled with large-scale functional brain networks remains largely unexplored.

Building on the limitations of previous studies and the advantages of RSA-based cross-modal analysis, the present study applied an EEG–fMRI multimodal representational similarity analysis framework. Using the Schaefer 2018 functional network parcellation atlas [23,24] as the spatial reference, we systematically investigated the representational dynamics of seven large-scale functional networks during VI, including the visual network (VIS), dorsal attention network (DAN), ventral attention network (VAN), frontoparietal network (FPN), default mode network (DMN), somatomotor network (SMN), and limbic network (LIM). Specifically, this study first integrated the biologically inspired CORnet-S visual model and mapped hierarchical model features onto brain network responses through representational similarity analysis to characterize the hierarchical organization of VI-related neural representations. In parallel, cross-modal analyses between multiband EEG temporal representations and fMRI-derived network representations were performed to investigate the spatiotemporal evolution of neural dynamics during VI. The primary goal of this study was to quantitatively evaluate the sensitivity and temporal specificity of large-scale functional networks to endogenous visual representations during VI. The findings of this study are expected to advance our understanding of how large-scale brain networks represent internally generated cognitive processes and to refine the spatiotemporal network mechanisms underlying top-down processing. Furthermore, these results may provide a network-level feature selection framework for VI-based brain–computer interfaces and offer insights into the pathological mechanisms of aberrant imagery in psychiatric disorders, as well as potential neural targets for imagery-based psychological interventions.

## 2. Materials and Methods

### 2.1. Participants and Ethics Statement

Participants were recruited through an open campus-wide recruitment procedure. A total of 13 participants (8 males and 5 females; mean age ± SD = 23.44 ± 1.51 years) were enrolled in the study. All participants were right-handed and had normal or corrected-to-normal visual acuity, normal color vision, and no history of visual impairment. Participants were excluded if they had a history of neurological or psychiatric disorders, traumatic brain injury, long-term use of neuropsychiatric medications, or any contraindications for MRI examination, including metallic implants or cardiac pacemakers. All participants exhibited intact cognitive function and were able to accurately understand the experimental instructions and complete all experimental procedures. All participants completed the Vividness of Visual Imagery Questionnaire (VVIQ), a measure previously associated with individual differences in neural mechanisms [25]. The mean VVIQ score was 62.38 ± 11.21. This study was conducted in accordance with the ethical principles for human research outlined in the Declaration of Helsinki by the World Medical Association. The experimental protocol was reviewed and approved by the Medical Ethics Committee of Kunming University of Science and Technology (approval number: KMUST-MEC-2023-029). Before participating in the experiment, all participants were fully informed of the study objectives, experimental procedures, potential risks, and their rights as research participants. Written informed consent was obtained from all participants prior to the experiment. Participants were informed that they could withdraw from the study at any time without providing a reason and without any loss of their legal rights.

### 2.2. EEG Data Acquisition and Preprocessing

#### 2.2.1. EEG Experimental Paradigm

The experimental stimuli are illustrated in Figure 1. The stimulus set consisted of commonly encountered objects, animals, and geometric shapes from daily life. Identical stimuli were used for both EEG and fMRI experiments. During the experiment, participants were instructed to recall the presented images in the form of visual representations rather than verbal descriptions or word-based lists. The EEG experimental paradigm is shown in Figure 2a. Each of the 10 stimulus images was presented 40 times, yielding a total of 400 trials. Each trial lasted 17 s and followed a fixed sequence. Each trial began with a 3 s fixation period, during which the word “Fixation” was presented at the center of the screen to instruct participants to maintain attention while remaining relaxed. Subsequently, a task-related image was randomly presented for 4 s, during which participants were instructed to observe and memorize the image. After image offset, a 2 s visual mask was presented to eliminate potential visual afterimages. The screen then turned black, indicating the onset of the VI period, during which participants performed visual imagery of the previously presented image for 4 s. Finally, a 4 s “Rest” prompt was displayed, during which participants were instructed to terminate imagery and relax. Throughout the experiment, no irrelevant auditory or visual stimuli were presented. Participants were required to keep their eyes open during the imagery period.

#### 2.2.2. EEG Data Acquisition

EEG data were acquired using a W3 32-channel EEG recording system (Neuracle, Hangzhou, China). The recording montage consisted of 32 scalp electrodes positioned according to the international 10–20 electrode placement system, including Fpz, Fp1, Fp2, Fz, F3, F4, F7, F8, FCz, FC3, FC4, FT7, FT8, Cz, C3, C4, T7, T8, CP3, CP4, TP7, TP8, Pz, P3, P4, P7, P8, PO3, PO4, Oz, O1, and O2. During EEG acquisition, CPz was used as the reference electrode (excluded from subsequent analyses), and AFz served as the ground electrode. EEG signals were recorded at a sampling rate of 1000 Hz. Before the experiment, the impedance of all electrodes was maintained below 5 kΩ. The spatial distribution of electrode locations is illustrated in Figure 3.

#### 2.2.3. EEG Data Preprocessing

The EEG preprocessing procedure was performed as follows: First, continuous raw EEG recordings were visually inspected, and bad channels were identified and marked for subsequent interpolation. The data were then filtered using a 50 Hz notch filter to remove power-line interference, followed by a 1–100 Hz zero-phase finite impulse response (FIR) band-pass filter to eliminate baseline drift and high-frequency environmental noise. After filtering, the data were re-referenced to the common average reference across all channels. To effectively remove physiological artifacts, independent component analysis (ICA) was performed using the Infomax algorithm. Artifact-related components, including ocular, cardiac, and myogenic artifacts, were identified and removed based on their spatial topographies and temporal characteristics. After artifact correction, previously identified bad channels were reconstructed using spherical spline interpolation. The data were subsequently filtered with an 8–80 Hz zero-phase FIR band-pass filter to isolate the frequency range of interest. Continuous EEG data were then segmented according to experimental event markers, and epochs corresponding to 0–4 s after task onset were extracted. Finally, the data were downsampled to 250 Hz to reduce computational redundancy while preserving relevant neural information.

### 2.3. fMRI Data Acquisition and Preprocessing

#### 2.3.1. fMRI Experimental Paradigm

The fMRI experiment used the same stimuli and task paradigm as the EEG experiment. The fMRI paradigm adopted a block design, as illustrated in Figure 2b, and EEG and fMRI data were acquired separately. Given the relatively slow hemodynamic response underlying the BOLD signal, a block design was employed in the fMRI experiment to extend the duration of the imagery period, thereby enhancing the detection of sustained BOLD responses. In contrast, the EEG experiment was designed to capture transient neural responses with high temporal resolution. The formal scanning session consisted of five independent scanning runs, each lasting approximately 12 min, resulting in a total scanning duration of approximately 1.2 h. A 2–4 min rest interval was provided between adjacent runs to reduce participant fatigue. Each block followed a fixed sequence. First, a 12 s baseline period was presented. After the baseline phase, participants viewed the experimental stimulus for 16 s, followed by a 16 s rest period. Participants then performed a 16 s VI task, during which they were instructed to mentally reconstruct the previously presented stimulus. After the imagery period, an additional 16 s rest period was included, completing one full block. Within each scanning run, each image was presented once, and the presentation order was pseudo-randomized to minimize potential order effects. Across the five scanning runs, each participant completed five imagery trials for each stimulus.

#### 2.3.2. fMRI Data Acquisition

Structural MRI acquisition: High-resolution anatomical images were acquired using a three-dimensional magnetization-prepared rapid gradient echo (3D-MPRAGE) sequence. The acquisition parameters were as follows: repetition time (TR) = 2300 ms; echo time (TE) = 2.29 ms; flip angle = 8°; field of view (FOV) = 240 × 240 mm^2^; matrix size = 256 × 256; slice thickness = 0.94 mm; and 192 sagittal slices. The high-resolution structural images were used for subsequent spatial registration and normalization of functional images. Functional MRI acquisition: Functional images were acquired using an echo-planar imaging (EPI) sequence. The acquisition parameters were as follows: TR = 2000 ms; TE = 30 ms; flip angle = 90°; FOV = 220 × 220 mm^2^; matrix size = 64 × 64; and slice thickness = 3.2 mm, with no interslice gap. Each functional volume consisted of 44 axial slices covering the whole brain, with a voxel size of 3.4 × 3.4 × 3.2 mm^3^.

#### 2.3.3. fMRI Data Preprocessing

The fMRI preprocessing procedure was performed as follows: First, slice timing correction was applied to the functional images to correct for temporal offsets caused by differences in acquisition time across slices. Subsequently, head motion correction was performed to reduce motion-related artifacts introduced during scanning. The functional images were then co-registered to each participant’s high-resolution T1-weighted structural image, followed by segmentation of the structural images into gray matter, white matter, and cerebrospinal fluid tissues. During the segmentation process, nonlinear deformation parameters from individual anatomical space to the standard Montreal Neurological Institute (MNI) space were estimated. The estimated deformation parameters, combined with the aforementioned co-registration information, were used to normalize all functional images into MNI space. Finally, the normalized functional images were spatially smoothed using a 6 mm full-width at half-maximum (FWHM) Gaussian kernel to improve the signal-to-noise ratio.

### 2.4. Definition of Functional Networks

This study employed the Schaefer 2018 [24] functional network parcellation atlas as the basis for subsequent analyses. The atlas was developed by Schaefer et al. using large-scale resting-state functional MRI datasets, in which the cerebral cortex was parcellated into functionally homogeneous regions of interest (ROIs) based on functional connectivity patterns and further assigned to the seven-network framework proposed by Yeo et al. It represents one of the most widely used and extensively validated cortical parcellation schemes in contemporary human brain functional research. The Schaefer 2018 atlas divides the cerebral cortex into seven large-scale functional networks characterized by highly consistent connectivity profiles: the visual network, somatomotor network, limbic network, frontoparietal network, default mode network, ventral attention network, and dorsal attention network. The atlas demonstrates strong reproducibility and generalizability, with stable network organization patterns observed across different participant populations and imaging acquisition parameters. These seven networks collectively span a broad functional hierarchy, ranging from primary sensory processing to higher-order cognitive control, thereby providing a comprehensive framework for characterizing functional specialization and interactions among large-scale brain systems. Importantly, this network parcellation is not limited to resting-state data; rather, its network activation patterns and connectivity profiles have also demonstrated robustness in task-based fMRI paradigms, supporting the investigation and interpretation of task-related cognitive processes. Visual imagery is not governed by a single visual cortical region but instead relies on the coordinated engagement of multiple functional systems, including visual, attentional, memory-related, and executive control networks. Therefore, based on the Yeo seven-network framework implemented in the Schaefer 2018 atlas, the present study investigated whole-brain representational patterns of VI at the network level, providing a macroscale framework for elucidating the higher-order cognitive architecture underlying visual imagery.

### 2.5. Representational Similarity Analysis

Representational similarity analysis is a computational framework in cognitive neuroscience that quantifies and compares the correspondence between the geometric structures of representational spaces across different data sources, including neuroimaging data, behavioral measurements, and computational models [26,27,28]. The analytical procedure of RSA follows a core logical pipeline consisting of four major steps: representation extraction, representational dissimilarity matrix (RDM) construction, cross-source matrix comparison, and statistical inference. First, based on the fundamental assumption that stimuli or experimental conditions with similar representational content should elicit more similar response patterns, multidimensional representation vectors corresponding to each experimental condition are extracted from the target data source. Subsequently, a selected dissimilarity metric is applied to quantify pairwise differences between all conditions, generating an N × N RDM, in which each off-diagonal element represents the degree of representational difference between a pair of conditions. Next, appropriate similarity or association measures are employed to quantify the correspondence between RDMs derived from different sources. Finally, statistical testing and multiple-comparison correction procedures are applied to evaluate the significance of the observed relationships, enabling quantitative inference regarding neural coding mechanisms and representational correspondence across modalities, species, and computational models. The overall RSA workflow is illustrated in Figure 4. RSA enables comparisons across measurement modalities and computational models by expressing each representation as an RDM whose entries describe pairwise dissimilarities among the same stimuli. Rather than comparing raw signal amplitudes or requiring a direct correspondence between EEG channels, fMRI voxels, and model units, RSA compares the representational relationships among stimuli. In this study, this framework was used to assess correspondence between CORnet-S and fMRI representations and, separately, between time-resolved EEG and network-specific fMRI representations. As EEG and fMRI represent distinct modalities of neural measurements, substantial differences exist in their signal dimensions, response characteristics, and quantification schemes, making direct comparison and integration challenging. Therefore, the present study compared them at the RDM level.

For the EEG data, time–frequency decomposition was performed using Morlet wavelets to estimate oscillatory power across different frequencies and time points. To address the inherent edge effects associated with wavelet transformation, mirror-symmetric signal extension was applied to the boundaries of each epoch to reduce truncation artifacts. Logarithmically transformed time–frequency power within the alpha (8–13 Hz), beta (13–30 Hz), and gamma (30–80 Hz) bands during the imagery period was then used to calculate Pearson correlation distances, generating frequency-specific EEG RDMs. EEG data were analyzed using a window length of 200 ms and a step size of 100 ms. Participant-level EEG RDMs were subsequently averaged to obtain group-level EEG RDMs for each frequency band and time window. For the fMRI data, a general linear model (GLM) [29,30] was first applied to model task-related responses, generating beta-weight maps for each experimental stimulus across all scanning runs. The Schaefer 2018 functional network parcellation atlas was then used to define network-level regions, from which voxel-wise response patterns within each functional network were extracted. Pearson correlation distances were subsequently calculated to construct network-specific RDMs. For cross-modal RSA between EEG and fMRI, each participant’s network-specific fMRI RDMs were correlated with the group-level EEG RDMs using Spearman correlation coefficients. Spearman correlation was used for all second-order RDM comparisons to quantify rank-based correspondence between representational dissimilarities without assuming a linear relationship between them.

### 2.6. CORnet-S Model and Hierarchical Feature Extraction

To investigate neural response patterns across different hierarchical levels of the brain during VI, the present study employed the CORnet-S model to extract hierarchical features from the visual stimuli [31]. CORnet-S is a shallow recurrent neural network whose architecture consists of four anatomically motivated stages, including V1, V2, V4, and IT, corresponding to four key regions along the primate ventral visual pathway. Unlike most purely feedforward deep neural networks, CORnet-S incorporates recurrent connections within the IT, V2, and V4 stages, enabling a better approximation of the recurrent and dynamic neural activity patterns involved in biological visual processing. Compared with conventional deep convolutional neural network architectures, CORnet-S exhibits a hierarchical architecture that more closely corresponds to the anatomical organization of the primate ventral visual cortex, making it particularly suitable for visual neuroscience investigations.

The architecture and block-level recurrent structure of the CORnet-S model are illustrated in Figure 5 (redrawn from the original publication). The fundamental computational unit of CORnet-S uses a ResNet-like bottleneck architecture. Within each block, an “expand–compress” convolutional design is employed to enhance feature representation capacity, while skip connections facilitate gradient propagation and information transmission. Importantly, recurrent connections are introduced within these blocks, allowing information to be iteratively processed over time and thereby providing a more biologically plausible approximation of neural dynamics within visual cortical regions. In the present study, a pretrained CORnet-S model was used to extract hierarchical features from visual stimuli. Specifically, feature representations were extracted from the V1, V2, V4, and IT stages, and layer-specific RDMs were constructed to characterize the representational structure across different levels of the model. Subsequently, the RDMs derived from each model stage were compared with fMRI-based RDMs of each functional brain network during both VP and VI tasks, enabling the evaluation of the correspondence between computational visual features and neural representations across brain networks.

To examine whether model–brain representational correspondences differ across functional networks as a function of task, we performed a task × network interaction analysis for each CORnet-S layer. For each layer, we computed the VIS–FPN representational difference and tested the contrast:

(VP_VIS_ − VP_FPN_) − (VI_VIS_ − VI_FPN_) (1)

which indexes the relative task-dependent shift in network-specific representational advantage. A positive interaction indicates that the VIS–FPN contrast is larger during VP than during VI. RSA coefficients were Fisher *z*-transformed prior to analysis. Statistical inference was conducted using a two-sided exact sign-flip test with 13 subjects, enumerating 8192 sign patterns. For each planned contrast, *p*-values were false discovery rates (FDRs) corrected across the four CORnet-S layers using the Benjamini–Hochberg procedure.

### 2.7. Statistical Analysis and Multiple Comparison Correction

Whole-brain activation analyses were conducted using a hierarchical statistical inference procedure combining an uncorrected voxel-level threshold with cluster-level multiple-comparison correction. Specifically, an uncorrected voxel-wise threshold of *p* < 0.005 was first applied to define candidate activated voxels and generate contiguous activation clusters. Subsequently, all identified clusters were subjected to whole-brain cluster-level FDR correction [32], and clusters surviving a corrected threshold of *q* < 0.05 were considered statistically significant. Throughout the manuscript, *q* denotes the FDR-adjusted *p*-value. For the representational similarity analysis between CORnet-S and fMRI data, Spearman correlations were calculated for each participant between RDMs derived from different CORnet-S layers and network-specific fMRI RDMs. The resulting correlation coefficients were then transformed using Fisher *z*-transformation [33]. At the group level, statistical significance was assessed using a two-sided sign-flip test on Fisher z-transformed RSA coefficients. Multiple comparisons across CORnet-S layers were controlled using the Benjamini–Hochberg FDR procedure, and *q* < 0.05 was considered statistically significant. VVIQ scores were correlated with the mean contrast estimates within a group-level significant mask derived from the VI > rest contrast. For each participant, the mean contrast estimate within the mask (including all significant clusters) was extracted from the corresponding first-level VI > rest contrast image. The association between VVIQ scores and the extracted contrast estimates was assessed using Spearman rank correlation with a two-sided Monte Carlo label-permutation test based on 10,000 random permutations of VVIQ scores across participants.

For the EEG–fMRI cross-modal representational similarity analysis, a cluster-based nonparametric permutation test was employed to identify significant temporal effects [34,35], combined with FDR correction across functional networks to control for multiple comparisons at the network level. Spearman correlation coefficients were calculated at each time window between the EEG RDM and the RDM of each functional network derived from each individual fMRI participant, followed by Fisher *z*-transformation. For each frequency band and functional network, one-sample t-tests were conducted to determine whether RSA values were significantly greater than zero (one-tailed test, with the expected direction being positive correlations). An uncorrected threshold of *p* < 0.1 was used as the cluster-forming threshold, and temporally adjacent significant time points were grouped into candidate clusters. The cluster statistic was defined as the sum of *t*-values within each temporally contiguous cluster. A null distribution was generated through 10,000 sign-flip permutations. In each permutation, all temporal clusters were extracted, and the maximum cluster statistic was recorded for that permutation. Cluster-level *p*-values were obtained by comparing the observed cluster statistics against the permutation-derived null distribution, and clusters with cluster-level *p* < 0.05 were considered statistically significant. For each frequency band, the *p*-value of the strongest temporal cluster within each functional network was entered into the Benjamini–Hochberg FDR procedure to correct for multiple comparisons across networks. The resulting *q* values were evaluated against a threshold of 0.05 and used to identify networks with significant temporal representational correspondence. BH-FDR correction was retained as the primary approach to multiple-comparison correction. As a supplementary sensitivity analysis, Bonferroni correction was applied to the same comparison families: the seven network-level *p*-values within each frequency band for EEG–fMRI RSA; the four layer-specific *p*-values within each task and functional network for CORnet-S–fMRI RSA; and the four layer-specific *p*-values for the task × network interaction.

A sensitivity power analysis was conducted to characterize the magnitude of participant-level effects detectable with the available sample. With *n* = 13, *α* = 0.05, and a target power of 0.80, the minimum detectable standardized effect size was approximately Cohen’s *d* = 0.847 for two-sided tests and *d* = 0.732 for one-sided tests. Thus, the present sample was primarily sensitive to relatively large participant-level effects. These values provide general benchmarks for the statistical sensitivity of the study rather than exact power estimates for each permutation- or cluster-based analysis.

## 3. Results

### 3.1. Brain Activation Patterns

To investigate the brain activation patterns associated with VP and VI, whole-brain activation analyses were conducted. Statistical inference for the whole-brain analysis employed a two-step correction strategy combining an uncorrected voxel-level threshold with cluster-level multiple-comparison correction. Specifically, an uncorrected voxel-wise threshold of *p* < 0.005 was first applied to identify candidate activated voxels and form spatially contiguous activation clusters. Subsequently, all whole-brain clusters were subjected to cluster-level false discovery rate correction for multiple comparisons, and clusters surviving the corrected threshold of *q* < 0.05 were reported as statistically significant activations.

#### 3.1.1. Activation Patterns During Visual Perception

The VP task revealed five significant activation clusters. The number of voxels, peak MNI coordinates, peak anatomical regions, and peak *t*-values of each cluster are summarized in Table 1. The activated regions were primarily distributed across the occipital visual cortex, thalamus, prefrontal cortex, and precentral gyrus. Two independent activation clusters were identified in the thalamus, showing bilateral activation, whereas peak activations in the occipital visual cortex, prefrontal cortex, and precentral gyrus were restricted to one hemisphere. Cluster 1 was the largest activation cluster, comprising 18,068 voxels, with the peak coordinate located at (−32, −72, −12). The peak activation was localized to the left fusiform gyrus, with a peak *t*-value of 12.326. Cluster 2 contained 529 voxels, with a peak coordinate of (−20, −28, −2), and the peak activation was located in the left lateral geniculate nucleus (LGN) of the thalamus (*t* = 7.219). Cluster 3 comprised 780 voxels, with a peak coordinate of (22, −30, 0), and the peak activation was located in the right thalamus (*t* = 7.319). Cluster 4 included 1718 voxels, with a peak coordinate of (44, −4, 56), and the peak activation was localized to the right middle frontal gyrus (*t* = 6.452). Cluster 5 contained 949 voxels, with a peak coordinate of (−44, −4, 56), and the peak activation was located in the left precentral gyrus (*t* = 6.136).

Figure 6 presents the cortical activation rendering maps associated with the visual perception task. Significant activation clusters are displayed from six different views of the cortical surface, including left lateral, anterior, right lateral, superior, posterior, and inferior views. The color bar on the right indicates the gradient scale of activation intensity. The significant activations induced by the visual perception task exhibited a widespread bilateral distribution across the cerebral hemispheres. The majority of activated regions were concentrated in the bilateral occipital visual cortices, with additional prominent activation observed in the bilateral prefrontal cortex and precentral gyrus.

#### 3.1.2. Activation Patterns During Visual Imagery

The comparison of the VI task against rest revealed three significant activation clusters. The voxel counts, peak MNI coordinates, peak anatomical locations, and peak *t*-values of all clusters are summarized in Table 2. The activated regions were primarily located in the left inferior frontal gyrus, left supramarginal gyrus, and left supplementary motor area, exhibiting a clear left-hemisphere predominance. Cluster 1 comprised 801 voxels, with a peak coordinate of (−54, 6, 18), localized to the left inferior frontal gyrus pars opercularis, with a peak *t*-value of 5.151. Cluster 2 contained 653 voxels, with a peak coordinate of (−38, −44, 38), and the peak activation was located in the left supramarginal gyrus (inferior parietal lobule), with a peak *t*-value of 4.101. Cluster 3 consisted of 680 voxels, with a peak coordinate of (−2, 22, 46), and the peak activation was located in the left supplementary motor area, with a peak *t*-value of 4.176.

Figure 7 presents the inflated cortical surface rendering of brain activations during the VI task. The significant activation pattern showed a predominantly left-lateralized distribution, with no substantial activation observed in the right cortical hemisphere. The activation patterns were distributed across the prefrontal cortex, inferior parietal lobule, and medial motor-related cortex. The strongest activation was observed in the left inferior frontal gyrus, with additional activation clusters located in the left supramarginal gyrus and supplementary motor area.

This study investigated the relationship between VVIQ scores (range 16–80; higher scores correspond to greater visual imagery vividness) and brain activation strength evoked by the VI task. As shown in Figure 8, Spearman correlation analysis revealed no statistically significant association between the two variables (*p* = 0.619; *ρ* = 0.149), with the fitted regression line being approximately horizontal; brain activation strength did not exhibit a significant increasing or decreasing trend as VVIQ scores increased.

### 3.2. CORnet-S Hierarchical Representations and Mapping to Brain Networks

#### 3.2.1. Internal Representational Structure of CORnet-S Hierarchical Layers

Figure 9 illustrates the correlation structure among hierarchical feature representations extracted from different stages of the CORnet-S model. The V1 stage exhibited positive correlations with V2, V4, and IT, with Spearman correlation coefficients (*ρ*) of 0.565, 0.158, and 0.171, respectively. Further analysis revealed relatively weak correlations between V2 and V4 (*ρ* = 0.037) and between V2 and IT (*ρ* = 0.107), whereas the V4–IT relationship showed a negative correlation (*ρ* = −0.145).

#### 3.2.2. RSA Mapping Between CORnet-S Hierarchical Features and Functional Brain Networks

The present study further examined the correspondence between feature RDMs extracted from different CORnet-S stages and fMRI-based representations of human functional brain networks. As shown in Figure 10a, during the VP task, only the RDMs of the visual network exhibited significant correlations with all CORnet-S feature RDMs, whereas no other functional networks reached statistical significance. Specifically, the VIS network showed positive correlations with all CORnet-S stages, with the numerically largest mean correlation observed at the V2 stage (*ρ* = 0.168; *q* = 0.011), followed by the IT stage (*ρ* = 0.164; *q* = 0.011), V4 stage (*ρ* = 0.144; *q* = 0.011), and V1 stage (*ρ* = 0.139; *q* = 0.011). These findings indicate that during visual perception, the hierarchical visual features extracted by CORnet-S primarily correspond to the representational structure of the visual cortical network. As shown in Figure 10b, during VI, none of the seven functional networks, including the FPN, showed significant correlations with any CORnet-S stage after FDR correction (all *q* > 0.05). Thus, significant model–brain correspondence was detected in the VIS during VP but was not detected in any network during VI under the present analysis. As shown in Figure 10c, task × network interaction was significant at all four CORnet-S stages after FDR correction within the interaction contrast (V1: *q* = 0.009; V2, V4, and IT: *q* = 0.006). The group-mean VIS-FPN contrast in RSA coefficients was positive during VP and negative during VI, indicating that the VIS-FPN difference shifted in opposite directions across the two tasks. The interaction therefore reflects a relative shift from a more VIS-dominant pattern during VP to a less VIS-dominant pattern during VI. As a supplementary sensitivity analysis, we also applied Bonferroni correction to the same comparison families (full statistical results are shown in the Supplementary Materials, Tables S1–S5). The pattern of results was unchanged: during VP, the VIS network remained significantly correlated with all CORnet-S stages (V1: Bonferroni-adjusted *p* = 0.019; V2: *p* = 0.026; V4: *p* = 0.046; IT: *p* = 0.041); during VI, no network showed significant correspondence with any CORnet-S stage (all Bonferroni-adjusted *p* > 0.050); and the task × network interaction remained significant at all four stages (V1: *p* = 0.036; V2: *p* = 0.019; V4: *p* = 0.010; IT: *p* = 0.018).

### 3.3. Multiband EEG–fMRI Temporal Representational Dynamics

To investigate the temporal evolution of functional network representations during the VI task, we employed a multimodal integration framework and performed frequency-specific, time-resolved representational similarity analysis. Specifically, cross-modal representational correspondence was examined separately within the alpha (8–13 Hz), beta (13–30 Hz), and gamma (30–80 Hz) frequency bands, allowing us to characterize the temporal dynamics of EEG–fMRI representational alignment across different oscillatory components.

As shown in Figure 11a, within the alpha band, significant temporal clusters were observed in the FPN, DMN, VAN, and VIS, although the temporal dynamics differed across networks (*q* = 0.001, 0.016, 0.001, and 0.016, respectively). The FPN and VAN each exhibited a significant temporal cluster spanning 100–3900 ms, whereas the DMN showed two significant clusters spanning 100–2500 ms and 3100–3900 ms. Thus, the significant temporal windows of the FPN, DMN, and VAN showed substantial overlap. The FPN reached its peak correlation at 3900 ms (*ρ* = 0.114), whereas the VAN and DMN reached their peak correlations at 700 ms (*ρ* = 0.112 and *ρ* = 0.114, respectively). Within their respective significant time windows, the FPN, DMN, and VAN showed broadly similar fluctuations in correlation coefficients and visually comparable group-mean temporal trajectories. The VIS exhibited a significant temporal cluster spanning 200–3900 ms, reaching its peak correlation at 3700 ms (*ρ* = 0.075). The DAN showed relatively low correlation values across the time course, with only brief transient increases and no significant extended temporal cluster. Descriptively, the group-mean peak correlation values were numerically higher in the FPN, DMN, and VAN than in the VIS. No direct statistical comparison of peak values was performed across networks.

As shown in Figure 11b, within the beta band, significant temporal clusters were observed in the FPN, VAN, DMN, LIM, and VIS (*q* = 0.004, 0.004, 0.005, 0.040, and 0.021, respectively). The FPN, DMN, and VAN each yielded a significant temporal cluster spanning approximately 100–3900 ms. The LIM exhibited a significant temporal cluster from 100 to 2900 ms, whereas the VIS showed a significant temporal cluster from 700 to 3200 ms. The FPN reached its peak correlation at 1800 ms (*ρ* = 0.128), the DMN at 3900 ms (*ρ* = 0.110), the LIM at 1700 ms (*ρ* = 0.100), and the VAN at 1800 ms (*ρ* = 0.112). The VIS reached its peak correlation at 2300 ms (*ρ* = 0.071). The temporal trajectories of the correlation coefficients in the FPN, DMN, and VAN were broadly similar.

As shown in Figure 11c, within the gamma band, significant temporal clusters were observed in the FPN, DMN, VAN, and LIM (*q* = 0.011, 0.008, 0.011, and 0.045, respectively), with all four networks exhibiting significant clusters spanning 100–3900 ms. The DMN reached its peak correlation at 100 ms (*ρ* = 0.099), whereas the FPN reached its peak correlation at 3300 ms (*ρ* = 0.090). The LIM reached its peak correlation at 1900 ms (*ρ* = 0.122), while the VAN reached its peak correlation at 100 ms (*ρ* = 0.086). The FPN and DMN exhibited broadly similar temporal trajectories of representational correspondence.

In supplementary sensitivity analyses using Bonferroni correction across the seven networks within each frequency band, network-level EEG–fMRI representational correspondence remained significant in the FPN and VAN across all three frequency bands and in the DMN within the beta and gamma bands. The alpha-band DMN and VIS effects did not survive this correction (adjusted *p* = 0.0518 and 0.0623, respectively), nor did the beta-band LIM and VIS effects (adjusted *p* = 0.2002 and 0.0847, respectively) or the gamma-band LIM effect (adjusted *p* = 0.1792). Full statistical results are provided in the Supplementary Materials (Tables S6–S8).

## 4. Discussion

### 4.1. Brain Activation Pattern Analysis

#### 4.1.1. Activation Analysis of Visual Perception

The significant activations induced by the VP task were primarily distributed across the occipital visual cortex, thalamus, frontal cortex, and precentral gyrus, consistent with the canonical visual processing network involved in visual perception [36]. The largest activation cluster was located in the left lingual gyrus and fusiform gyrus, which constitute key components of the ventral visual pathway and are extensively involved in visual object recognition, shape processing, and semantic visual representations [37]. The extensive activation observed in the left lingual and fusiform gyri indicates that VP primarily relies on the ventral visual pathway for detailed representations of externally presented visual stimuli, consistent with previous findings in visual perception research. Meanwhile, significant activations were observed in the bilateral thalamus, particularly in the left LGN. Beyond its role in relaying visual information, the LGN receives cortical feedback modulation and contributes to visual selective attention and sensory gating processes [38]. Therefore, the bilateral thalamic activation observed in the present study not only reflects relay processing of visual information prior to cortical transmission but also suggests the presence of dynamic cortico-thalamic regulation during early visual processing. Furthermore, significant activations were identified in the right middle frontal gyrus and left precentral gyrus, suggesting that VP is not solely driven by bottom-up sensory input but also involves the engagement of higher-order cognitive systems. The middle frontal gyrus, as an important component of the frontoparietal network, is closely associated with attentional allocation, maintenance of task goals, and executive control. Its activation may reflect cognitive control processes engaged during the processing of externally presented visual stimuli [39]. Overall, the VP task exhibited a spatial organization characterized by coordinated activation of the thalamus, visual cortex, and frontal regions, indicating that visual perception involves not only canonical bottom-up sensory transmission but also sustained interaction with higher-order cognitive systems [40].

#### 4.1.2. Activation Analysis of Visual Imagery

The significant activations induced by the VI task were primarily concentrated in the left inferior frontal gyrus, left supramarginal gyrus, and left supplementary motor area, with an overall left-lateralized distribution. In contrast to the widespread activation of the occipital visual cortex and thalamus observed during the VP task, the VI task did not exhibit significant activation in early visual cortices. This activation pattern is consistent with the involvement of higher-order cognitive regions during VI [41]. However, the absence of significant early visual cortical activation at the applied threshold should not be interpreted as evidence that early visual regions were not involved. The left inferior frontal gyrus, a key region involved in language processing and working memory maintenance, showed significant activation, suggesting that VI may involve the retrieval of semantic features related to imagined objects and the sustained maintenance of internal visual representations [42]. Activation of the left supramarginal gyrus may reflect the internal simulation of object spatial information and the allocation of spatial attentional resources during imagery generation [43]. In addition, as shown in Figure 8, VI-related brain activation was not significantly associated with VVIQ scores. This non-significant association should be interpreted cautiously, as the relationship between self-reported imagery vividness and neural responses may be influenced by inter-individual variability and differences between questionnaire-based and task-specific measures of imagery. Previous studies have likewise suggested that VVIQ scores may not fully capture individual differences in task-specific imagery experience or neural responses during imagery [44]. Future studies could therefore incorporate complementary measures, such as post-imagery questionnaires and trial-by-trial responses, to better capture imagery-related subjective experience and strategy use.

As observed in Figure 6 and Figure 7, the two separately thresholded activation maps exhibited partially overlapping regions within the frontal cortex, suggesting that VP and VI may share certain cognitive neural mechanisms [45]. Both tasks involved activation in portions of the frontal cortex, indicating that cognitive control processes contribute to both externally driven visual perception and internally generated visual imagery, despite their different sources of visual information.

As shown in Figure 12, compared with the VI task, the VP task elicited a significant activation cluster comprising 20,980 voxels in the visual cortex. The specific information is presented in Table 3. This cluster was primarily distributed across the bilateral occipital lobes and encompassed key nodes of both the ventral and dorsal visual pathways, including the bilateral cuneus, lingual gyrus, calcarine cortex, middle occipital gyrus, and fusiform gyrus. These findings indicate that externally driven visual perception relies more heavily on bottom-up sensory processing pathways than internally generated visual imagery, engaging a hierarchical visual processing system extending from the primary visual cortex (Brodmann area 17) and secondary visual cortices (Brodmann area 18/19) to higher-order visual association cortices. The enhanced responses observed in occipital visual cortices during VP relative to VI suggest that these regions are more sensitive to perceptual processing of external visual inputs. However, this finding does not exclude the involvement of occipital visual regions during VI, as early visual cortices may still contribute to imagery processing through weaker or more distributed patterns of activation [46,47].

### 4.2. CORnet-S Hierarchical Mapping

The internal hierarchical correlations among CORnet-S representations (Figure 9) revealed that lower-level stages exhibited greater representational overlap, whereas higher-level stages showed more pronounced representational differentiation. This computational property closely parallels the hierarchical processing organization of the primate visual system, progressing from retinal inputs and the early visual cortices V1 and V2 to the higher-order IT cortex. The moderate positive correlation observed between lower-level stages (*ρ* = 0.565) reflects the gradual propagation of shared low-level visual features, such as edges and textures, across early processing stages. In contrast, the V4–IT relationship exhibited a weak negative correlation (*ρ* = −0.145), indicating increased representational divergence at higher hierarchical levels. This functional differentiation within the model provides a computational framework for investigating the neural mechanisms underlying VI.

As shown in Figure 10, during the VP task, CORnet-S, a model constructed based on the ventral visual pathway, exhibited hierarchical features that primarily captured the processing of externally presented visual inputs, and significant model–brain correspondence was detected in the VIS network. This model–brain correspondence is consistent with the hierarchical organization of the visual system, in which visual information is progressively transformed from lower-level visual regions to higher-order visual areas. Notably, the degree of correspondence between the VIS network and different CORnet-S stages varied, with the V2 stage showing the numerically largest mean correlation coefficient among the four model stages. This finding suggests that under the current visual task and stimulus conditions, intermediate-level representations within the visual cortex may exhibit greater similarity to the model feature space, rather than following a simple monotonic increase in correspondence along the model hierarchy. In contrast to VP, no network showed significant correspondence with CORnet-S representations after correction during the VI task. Because VI occurs in the absence of direct visual input, it is thought to depend on the interaction between memory representations, task goals, and higher-order cognitive systems that regulate internally generated visual representations. Although the FPN is thought to be a core network for VI and top-down control [48], its representations did not show significant correspondence with CORnet-S features. This dissociation is consistent with the view that CORnet-S was used as a computational model of stimulus-driven hierarchical visual processing but not the top-down, internally generated representations central to VI. Future visual models that integrate top-down feedback mechanisms, multimodal semantic representations, and cognitive control modules may provide a more comprehensive account of the complex neural representations underlying VI. The significant task × network interaction instead establishes a task-dependent change in the relative VIS–FPN contrast (Figure 10c). The contrast was more positive during VP than during VI, but this relative difference does not establish significant model correspondence in the FPN during VI or identify which individual network accounts for the interaction. The supported conclusion is therefore a change in the relative network profile of model correspondence, which is also compatible with top-down processing theories [49,50,51].

To further investigate the neural representational geometry underlying the fMRI–CORnet-S correspondence results, we visualized the RDMs of the visual network during both the VI and VP tasks (Figure 13). Using the VIS RDM of each participant, within-category and between-category distances were extracted based on three semantic categories of stimuli: animals, geometric shapes, and objects. As shown in Figure 13, the VP task exhibited clear category-based clustering in the RDM structure. Stimuli from the animal, geometric-shape, and object categories formed distinct clusters, indicating that neural activity patterns were more similar among stimuli within the same category. In contrast, no comparable clustering pattern was observed during the VI task, with representational patterns within each category appearing relatively dispersed. To quantitatively validate these observations, a one-sided sign-flip permutation test was performed to compare within-category and between-category distances, testing the directional hypothesis that within-category distances were smaller than between-category distances. The results showed that during VP, between-category distances were significantly larger than within-category distances (*p* = 0.007, Cohen’s *d* = 0.849), confirming that the category-based representational structure observed in the visualization was statistically significant. In contrast, during VI, no significant difference was observed between within-category and between-category distances (*p* = 0.930), and the direction of the effect was inconsistent, with the mean within-category distance being slightly larger than the mean between-category distance. These findings indicate that the visual network exhibited a clear category-based representational organization during VP, whereas such categorical clustering was not evident during VI.

Recent studies have increasingly demonstrated that VI does not simply reproduce perceptual experiences or perceptual representations. Using 7T fMRI, Bainbridge et al. (2021) showed that neural representations during visual recall differed from those established during encoding, both in representational structure and spatial localization, suggesting that recall is not merely a reactivation of encoding patterns [52]. Building on these findings, Breedlove et al. (2020) proposed a generative feedback model and demonstrated that feedback-driven processing results in substantially lower neural coding precision during imagery than during perception, with imagery representations characterized by larger receptive fields and coarser spatial frequency information [53]. Given the limited stimulus set employed in the present study, the absence of stable category-based clustering during VI may reflect the loss of fine-grained categorical information during top-down feedback transmission. Rather than representing a static retrieval of previous perceptual experiences or a simple reinstatement of perceptual representations, VI may involve a dynamic constructive process driven by feedback-based generative mechanisms.

### 4.3. Temporal Representational Differences of Multiband Neural Oscillations

In the present study, frequency-specific EEG–fMRI time-resolved representational similarity analysis was employed to investigate the network-level representational characteristics associated with different neural oscillatory bands during VI. Integrating the significant temporal clusters identified across the three frequency bands in Section 3.3, we found that alpha, beta, and gamma oscillations exhibited frequency-specific temporal dynamics and distinct patterns of network-level correspondence. These findings suggest that VI may rely on the differential involvement of multiple functional networks across distinct oscillatory frequencies.

#### 4.3.1. Alpha Band

As shown in Figure 11a, within the alpha band, the FPN, DMN, and VAN exhibited sustained and highly overlapping significant temporal clusters. The cross-modal correlation time series of these three networks showed similar dynamic trajectories, suggesting that multiple higher-order cognitive networks exhibit temporally aligned representational correspondence with the associated EEG-derived representations. Previous studies have suggested that alpha oscillations reflect not only cortical inhibitory processes but also contribute to the maintenance of internal attention, working memory retention, and endogenous information processing [54]. In the present study, the FPN and DMN both demonstrated prolonged significant temporal clusters, which may reflect the involvement of executive control and default-mode-related representations in maintaining internally generated information during VI. The representational correspondence observed in the VAN may be associated with attention-related processes engaged during imagery generation and maintenance [55,56]. The VIS also showed sustained alpha-band correspondence from 200 to 3900 ms, suggesting that visual-network representations remained involved alongside higher-order cognitive networks during imagery.

#### 4.3.2. Beta Band

As shown in Figure 11b, within the beta band, the FPN, DMN, and VAN exhibited relatively overlapping significant temporal clusters. This pattern indicates that the fMRI-derived representational structures of these networks showed similar temporal correspondence with beta-band neural representations. Previous studies have suggested that beta oscillations are involved in long-range cortico-cortical communication and the maintenance of cognitive states [57,58]. Therefore, the present findings may indicate that beta-band activity exhibits stable representational correspondence with information maintenance and integration processes supported by higher-order cognitive networks, while the significant correspondence observed in the VIS suggests that visual-network representations also contribute during this period [59].

#### 4.3.3. Gamma Band

Gamma oscillations are generally considered to reflect synchronized activity within local neuronal populations and have been implicated in visual feature integration, perceptual binding, and the maintenance of fine-grained visual representations [60]. As shown in Figure 11c, significant cross-modal representational correspondence was observed in the FPN, DMN, VAN, and LIM within the gamma band, with all four networks exhibiting significant temporal clusters spanning approximately 100–3900 ms. This pattern suggests that gamma-band representational correspondence is not restricted to a specific stage of visual imagery but instead persists across multiple higher-order functional networks throughout much of the imagery period [61]. In the supplementary Bonferroni analysis across networks, the FPN, DMN, and VAN effects remained significant, whereas the LIM effect did not (FDR-adjusted *q* = 0.045; Bonferroni-adjusted *p* = 0.179). The LIM reached its peak correlation at 1900 ms (*ρ* = 0.122). In the Schaefer seven-network parcellation used here, this network primarily includes regions such as the orbitofrontal cortex and temporal pole, which have been associated with contextual, semantic, and associative processing. The LIM correspondence observed under FDR correction may therefore be compatible with the involvement of such information in internally generated visual representations.

Across different frequency bands, the FPN and DMN exhibited relatively similar temporal trajectories, suggesting that these higher-order cognitive networks share comparable temporal correspondence with neural representations during VI [62,63]. The FPN showed extended significant temporal clusters across all three frequency bands, whereas significant VIS correspondence was detected in the alpha and beta bands but not in the gamma band. These findings suggest that VI-related representational correspondence involves both higher-order cognitive and visual networks. In the supplementary Bonferroni analysis, correspondence in the FPN and VAN remained significant across all three frequency bands, while DMN correspondence remained significant in the beta and gamma bands. However, the alpha-band DMN and VIS effects, the beta-band LIM and VIS effects, and the gamma-band LIM effect did not survive Bonferroni correction. These findings were therefore sensitive to the correction procedure, warranting caution in interpreting the specific functional contributions of the affected networks.

Taken together, the cross-modal RSA results across the three frequency bands demonstrate that multiple functional networks exhibit EEG-related temporal representational dynamics during VI, with frequency-specific patterns of network involvement. The alpha-band representational pattern may be associated with the regulation of internal attention and the maintenance of task-related cognitive states; the beta-band pattern may reflect cross-network representational coordination among distributed functional systems; and the gamma-band pattern may be more closely related to the maintenance and integration of fine-grained visual representations. Notably, the FPN exhibited significant cross-modal representational correspondence across all three frequency bands, suggesting sustained involvement of higher-order cognitive control networks in the representational processes underlying VI. This finding is consistent with theoretical models emphasizing the contribution of higher-order cognitive networks to imagery processing. However, it should be noted that RSA primarily characterizes similarity relationships between neural representations and cannot directly reveal the direction of information flow or causal interactions between functional networks. Therefore, the present findings indicate that higher-order cognitive networks and visual systems may engage in coordinated representational processes during VI. The frequency-dependent differences observed across bands further suggest that distinct oscillatory components may correspond to different aspects of neural representation during VI, highlighting the frequency-specific organization of imagery-related neural dynamics.

As shown in Figure 14, cross-modal correspondence patterns between fMRI representations and EEG oscillatory representations differed across the three frequency bands. Meanwhile, the temporal trajectories across the three frequency bands were not completely identical. The correspondence between FPN representations and the three EEG frequency bands exhibited distinct temporal patterns, suggesting that the FPN may support functionally differentiated processes across different oscillatory frequencies [64,65].

This study used fMRI RSA to characterize distributed neural representational similarity induced by visual imagery and further examined EEG-fMRI correlations as well as representational similarities between CORnet-S and fMRI. However, RSA captures representational patterns rather than time-varying directed signal transmission and thus cannot assess directionality. Nonparametric directionality analysis based on cross-correlation/coherence can resolve directed information flow between brain networks from EEG time series [66] and circumvents the model-order selection issues inherent in autoregressive-based directionality measures such as Granger causality. Future studies using such approaches may distinguish feedforward and feedback interactions among VIS, FPN, and DMN, complementing RSA findings at the representational level from a dynamic connectivity perspective.

### 4.4. Study Limitations and Future Directions

The present study has several limitations. First, the relatively small sample size may have reduced statistical power and limited the generalizability of the findings; sensitivity analysis indicated that the current sample was primarily sensitive to relatively large participant-level effects, whereas smaller effects may not have been reliably detected. Second, the study employed only a static VI paradigm and did not include dynamic imagery tasks, limiting inferences regarding brain interactions during dynamic imagery processes. Third, RSA characterizes representational correspondence but cannot directly establish causal interactions or information flow between brain regions. Fourth, although participants completed the VVIQ as a general measure of imagery vividness, no trial-by-trial or post-task ratings of imagery vividness, absorption, or task engagement were collected, limiting our ability to directly relate the observed neural representational patterns to participants’ subjective imagery experience during the task. Fifth, averaging EEG RDMs across participants attenuates individual differences in EEG representational geometry and temporal dynamics; although individual fMRI RDMs were retained, the cross-modal correlations reflect correspondence with a shared group-average EEG reference, thereby losing between-participant variability in EEG representations.

To address these limitations, future studies should pursue five corresponding directions. First, to increase statistical power and generalizability, larger and more diverse cohorts should be recruited, ideally guided by a priori power analyses, and the robustness of the present findings should be tested through replication and sensitivity analyses. Second, to overcome the limitation of a purely static VI paradigm, future work should incorporate dynamic imagery tasks and a broad range of imagery paradigms (e.g., object, scene, and motion imagery), enabling inferences about brain interactions during dynamic imagery processes. Third, to complement the correlational nature of RSA, directed or effective connectivity analyses should be incorporated to quantify causal interactions and information flow within top-down and bottom-up pathways. Fourth, to better capture subjective experience, trial-by-trial and post-task ratings of imagery vividness, absorption, and task engagement should be collected and related to neural representational patterns. Fifth, to preserve individual differences in EEG representational geometry and temporal dynamics, future studies may implement individual-specific EEG–fMRI RSA.

## 5. Conclusions

In this study, we employed EEG–fMRI cross-modal representational similarity analysis to investigate the neural representations underlying visual imagery from three complementary perspectives: spatial network organization, computational model mapping, and temporal dynamics. Activation analyses revealed that compared with visual perception, VI did not exhibit comparable responses in occipital visual cortices but instead showed a neural activity pattern primarily involving frontal, parietal, and medial cortical regions. These findings suggest that VI is not simply supported by the reactivation of sensory cortical representations but may involve the engagement of higher-order cognitive regions. CORnet-S-based analyses further revealed distinct model–brain representational correspondence between VP and VI. During VP, significant correspondence was predominantly observed within the visual network, with a significant task × network interaction indicating greater visual-network dominance relative to the frontoparietal network than during VI. In contrast, no significant CORnet-S correspondence was detected during VI, which may suggest distinct representational organizations for externally driven perception and internally generated imagery. EEG–fMRI cross-modal RSA further revealed frequency- and time-dependent representational correspondence across functional networks. The FPN, DMN, and VAN exhibited relatively sustained significant correspondence across multiple frequency bands, whereas the VIS and LIM displayed distinct temporal dynamics. Alpha and beta oscillations were primarily characterized by sustained correspondence with higher-order cognitive networks, whereas gamma oscillations involved broad representational relationships encompassing the frontoparietal, default mode, ventral attention, and LIM. Collectively, these findings demonstrate that VI is supported by a distributed, network- and frequency-dependent representational architecture, rather than being supported by a single brain region or frequency band. This study provides multimodal evidence for understanding the neural basis of VI and its representational mapping with computational models.

## Figures and Tables

**Figure 1:**
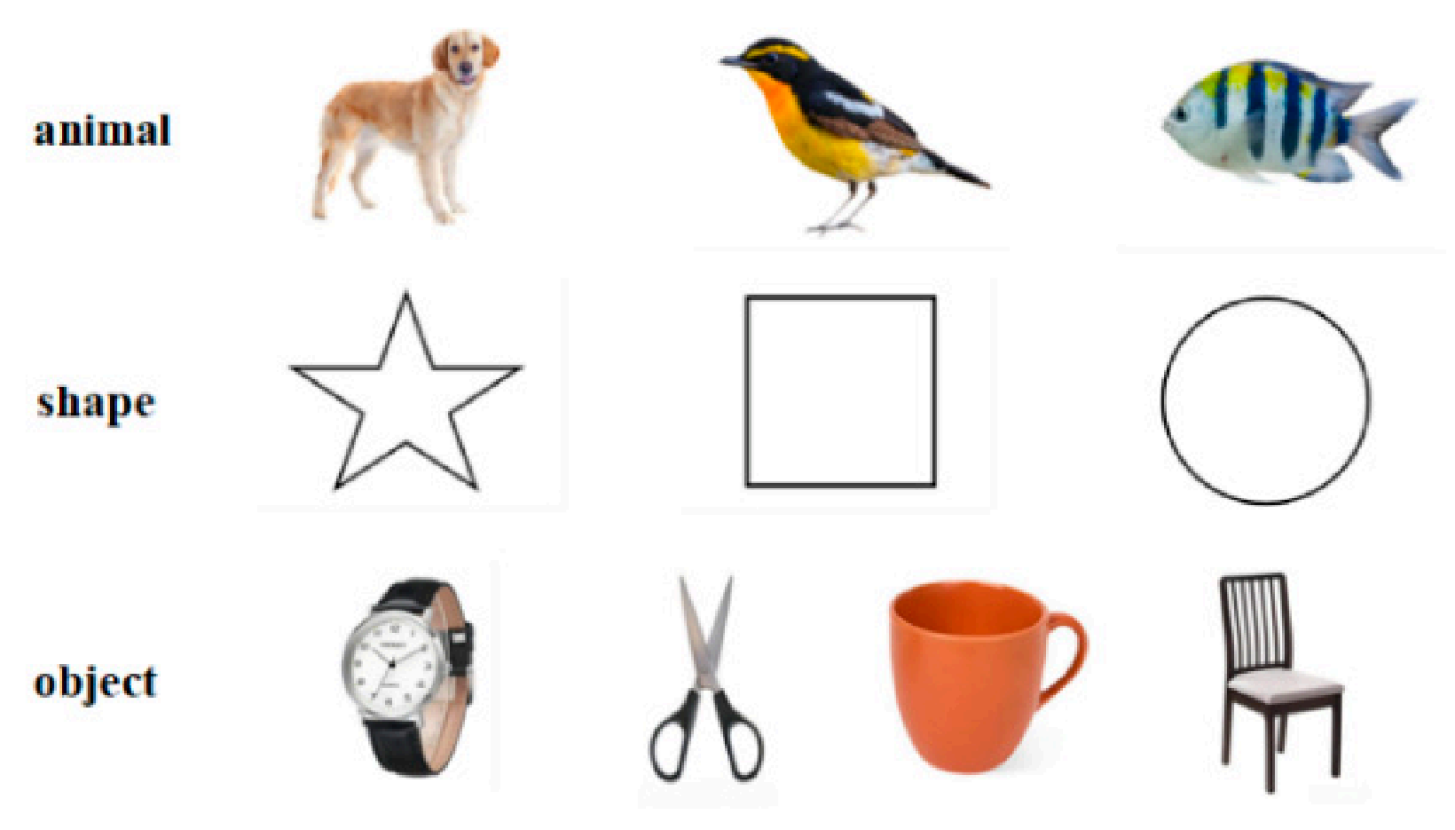
Experimental stimuli.

**Figure 2:**
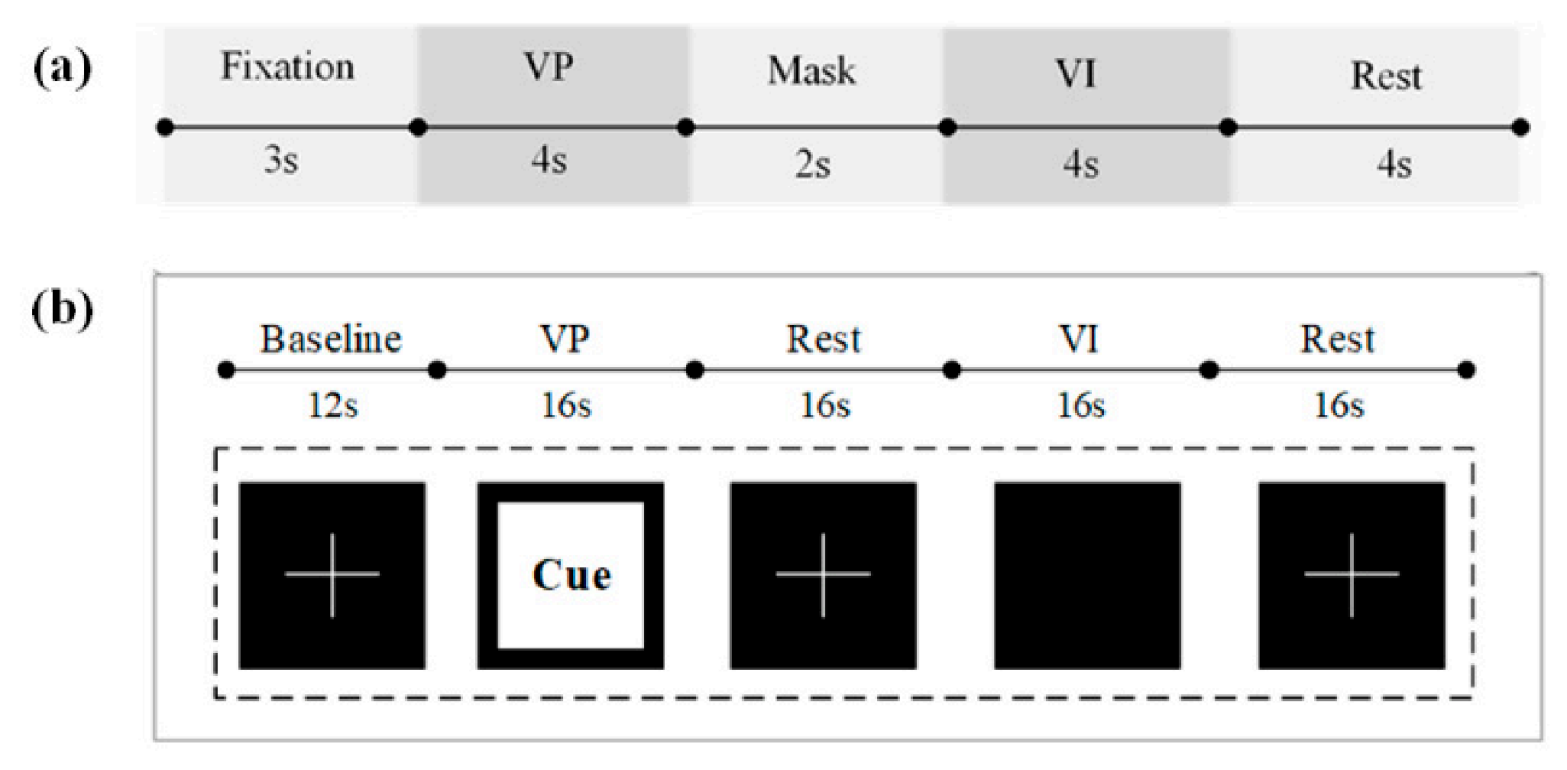
Experimental paradigms. (**a**) EEG experimental paradigm. (**b**) fMRI experimental paradigm.

**Figure 3:**
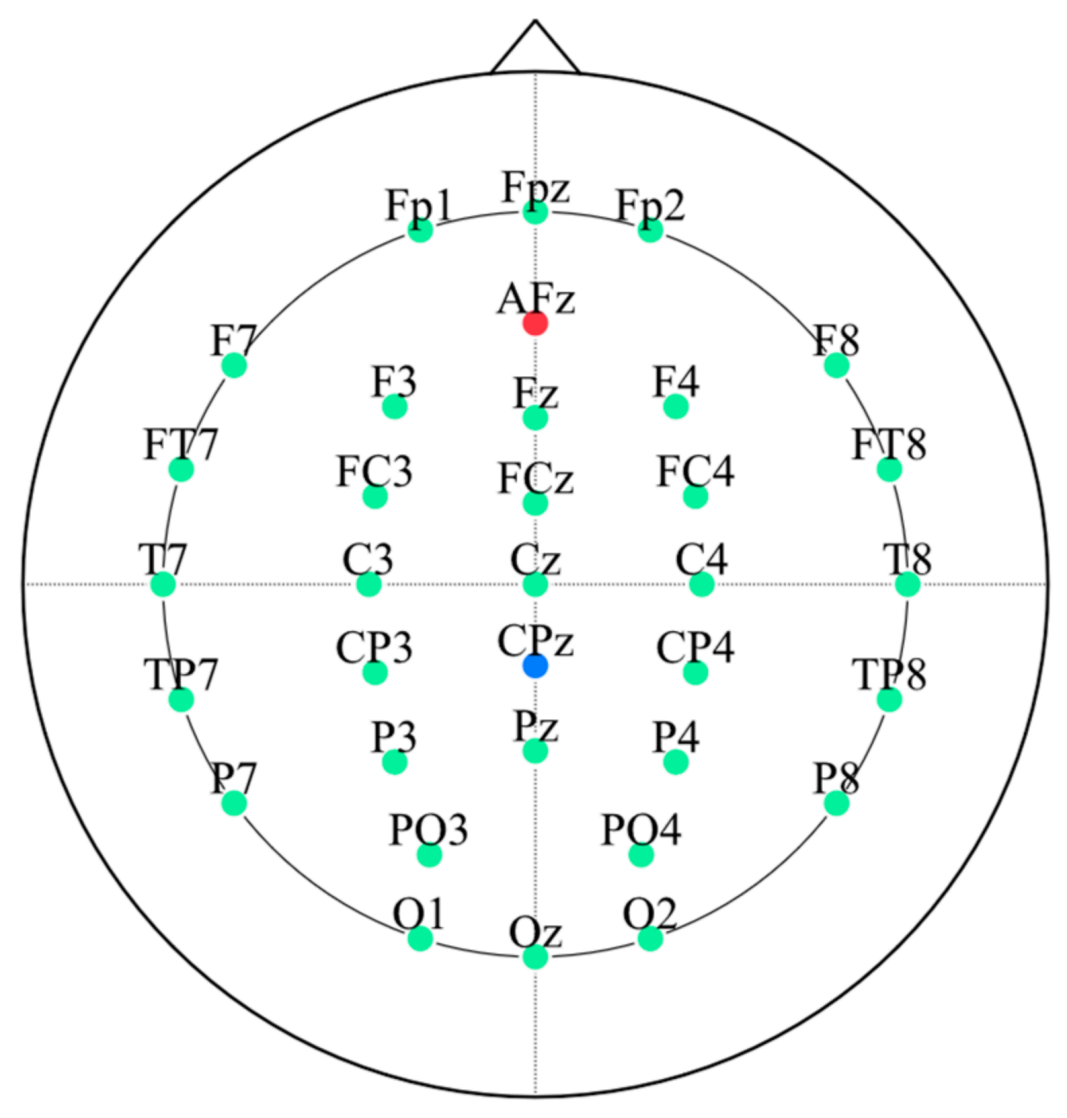
Electrode montage and channel distribution. Green dots represent standard EEG electrodes; the red and blue dots indicate AFz (ground) and CPz (reference), respectively. Dashed lines indicate the reference lines of the International 10–20 system.

**Figure 4:**
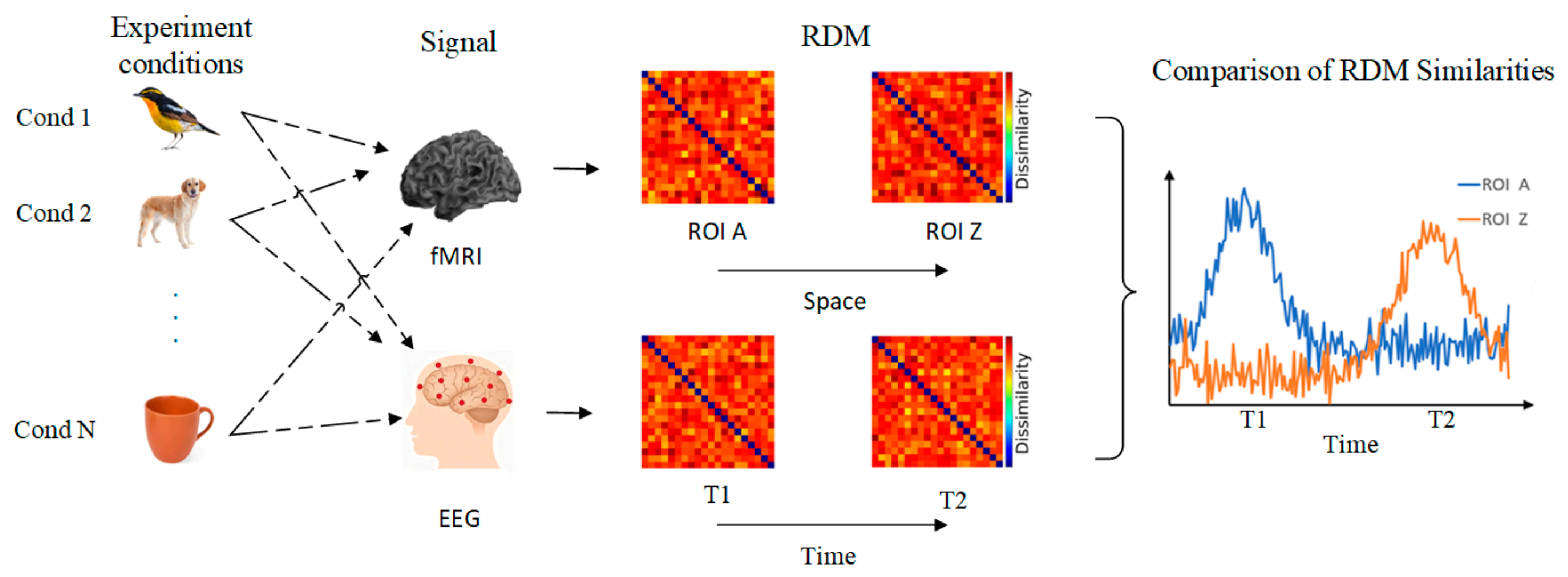
Schematic illustration of the representational similarity analysis workflow. Experimental conditions (Cond 1–N) were presented to participants, and the corresponding fMRI and EEG signals were recorded. Dashed arrows indicate that each experimental condition evokes neural responses, which are recorded as fMRI and EEG signals.

**Figure 5:**
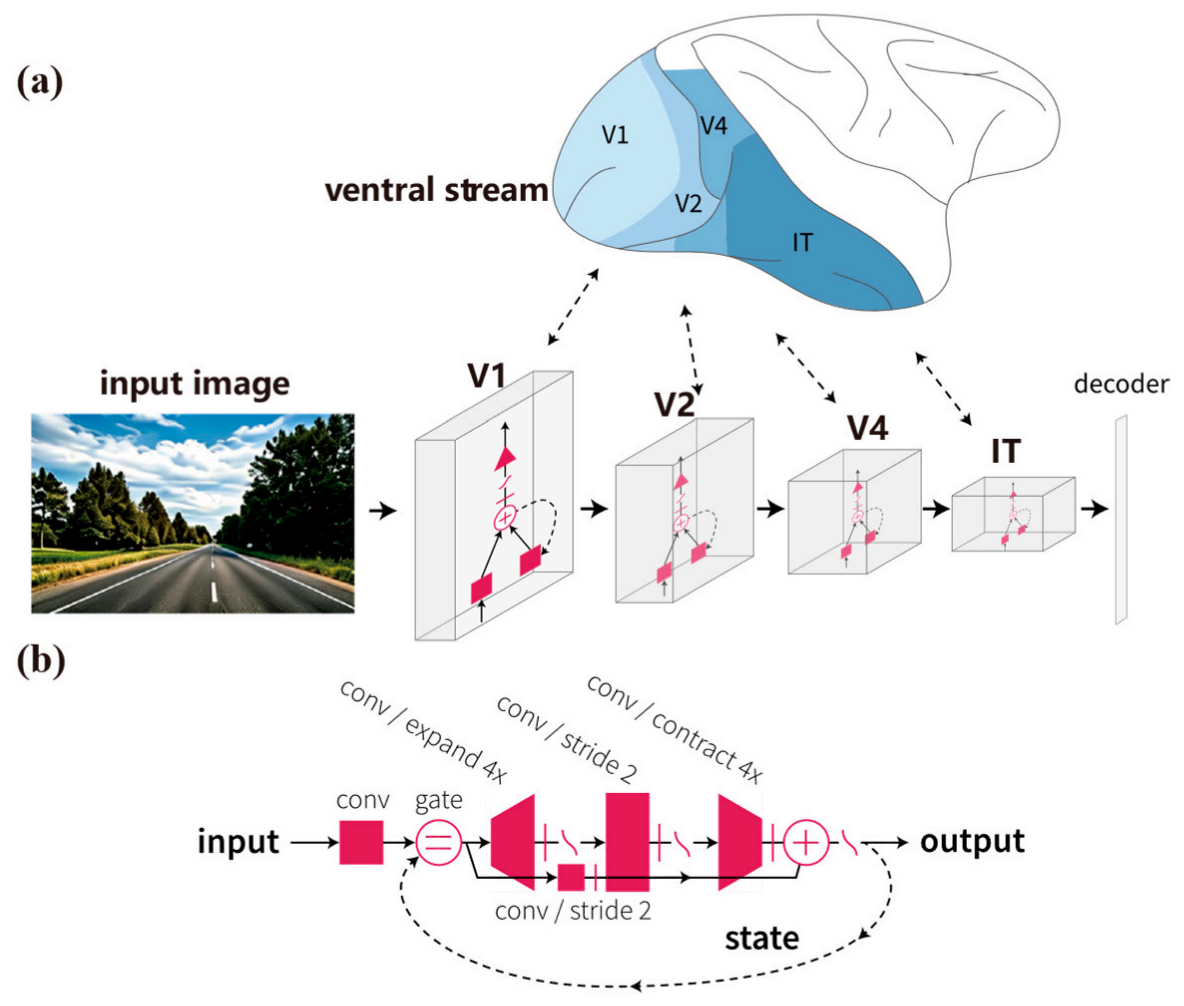
Architecture of the CORnet-S model. (**a**) The overall CORnet-S architecture comprises four computational modules: V1, V2, V4 and IT, corresponding to the primate ventral visual pathway. In the brain schematic, blue shading gradually darkens from V1 to IT to illustrate hierarchically increasing levels of visual processing. Solid arrows represent feedforward propagation of visual features across modules, while dashed arrows characterize the mapping between brain regions and model modules; (**b**) structure of the CORnet-S recurrent block. Red elements denote convolutional operations; solid arrows indicate intra-block feedforward computation, and the dashed loop labelled “state” represents recurrent state transfer, where the output of the previous time step serves as the input for the next time step.

**Figure 6:**
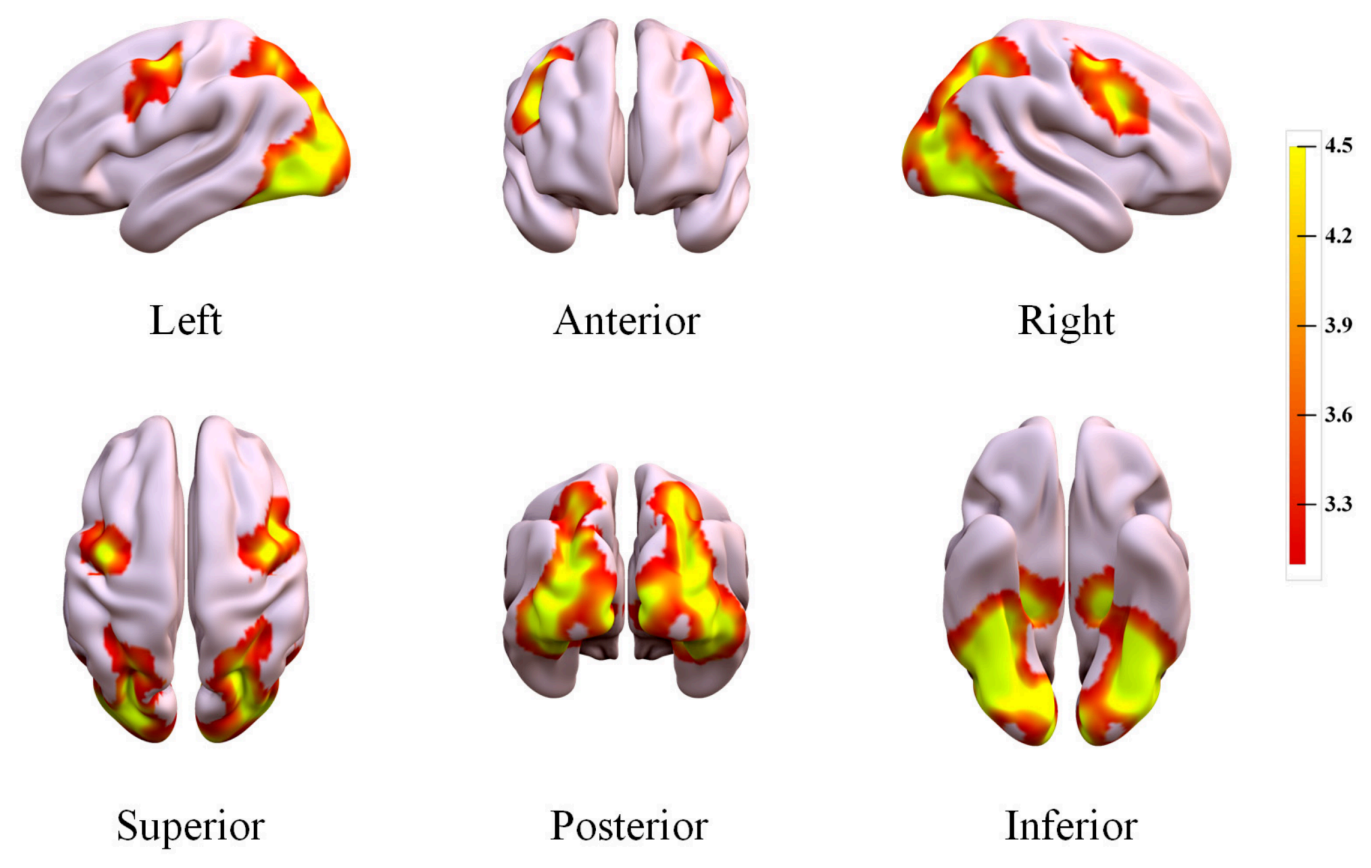
Activation patterns during the VP task. The color bar represents the gradient of rendered *t*-values.

**Figure 7:**
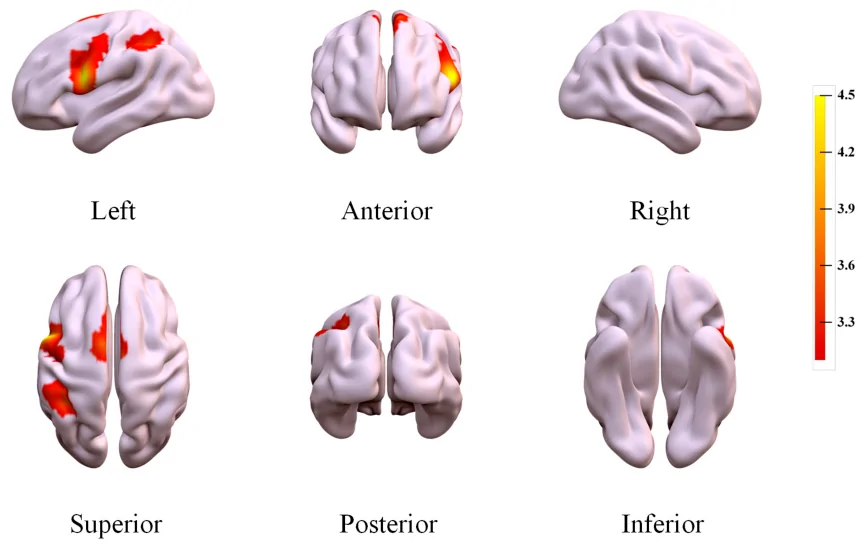
Activation patterns during the VI task. The color bar represents the gradient of rendered *t*-values.

**Figure 8:**
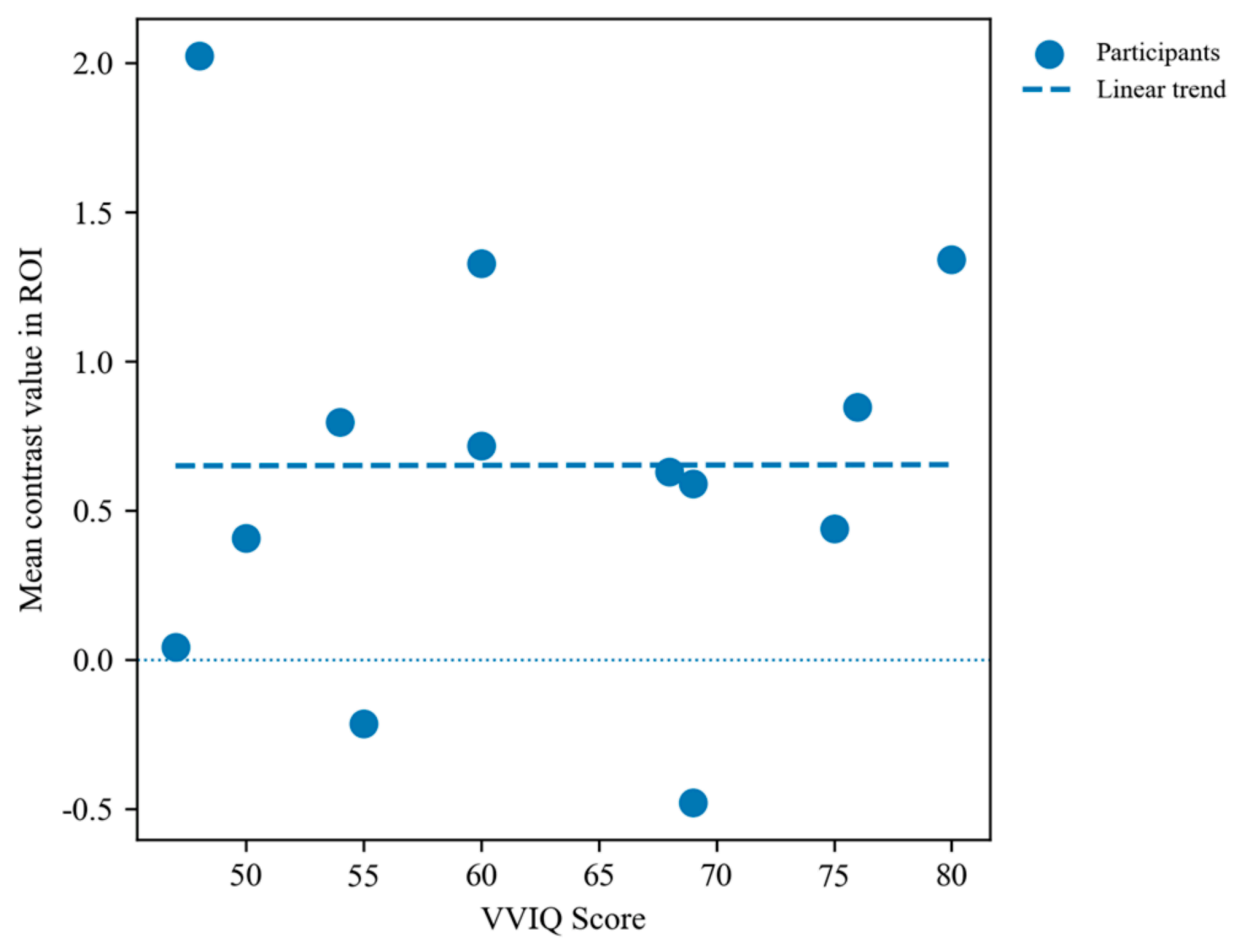
Scatter plot with linear fit for VVIQ scores versus mean contrast value in the ROI. Dashed line indicates the linear regression trend.

**Figure 9:**
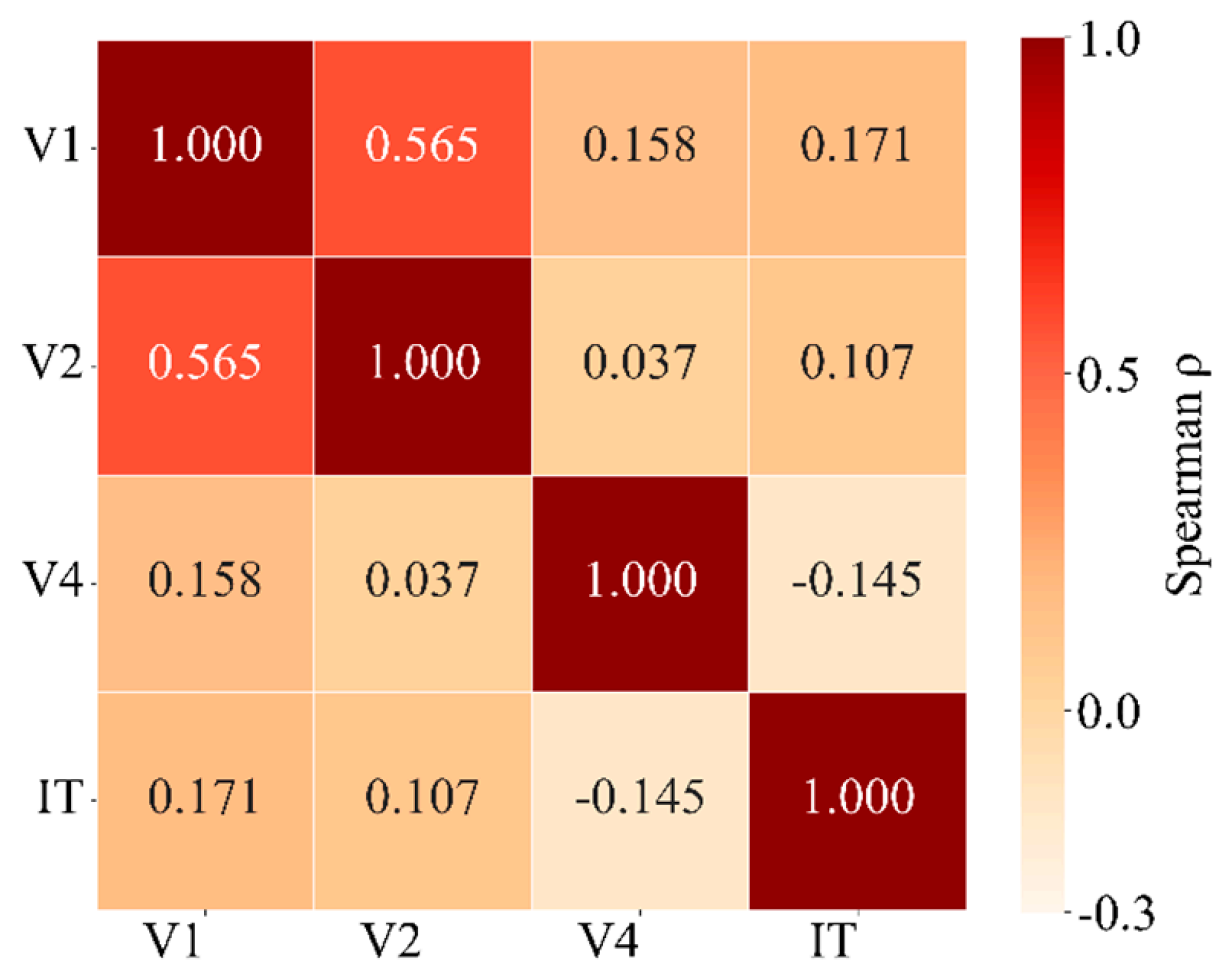
Representational similarity matrix among hierarchical stages within the CORnet-S model.

**Figure 10:**
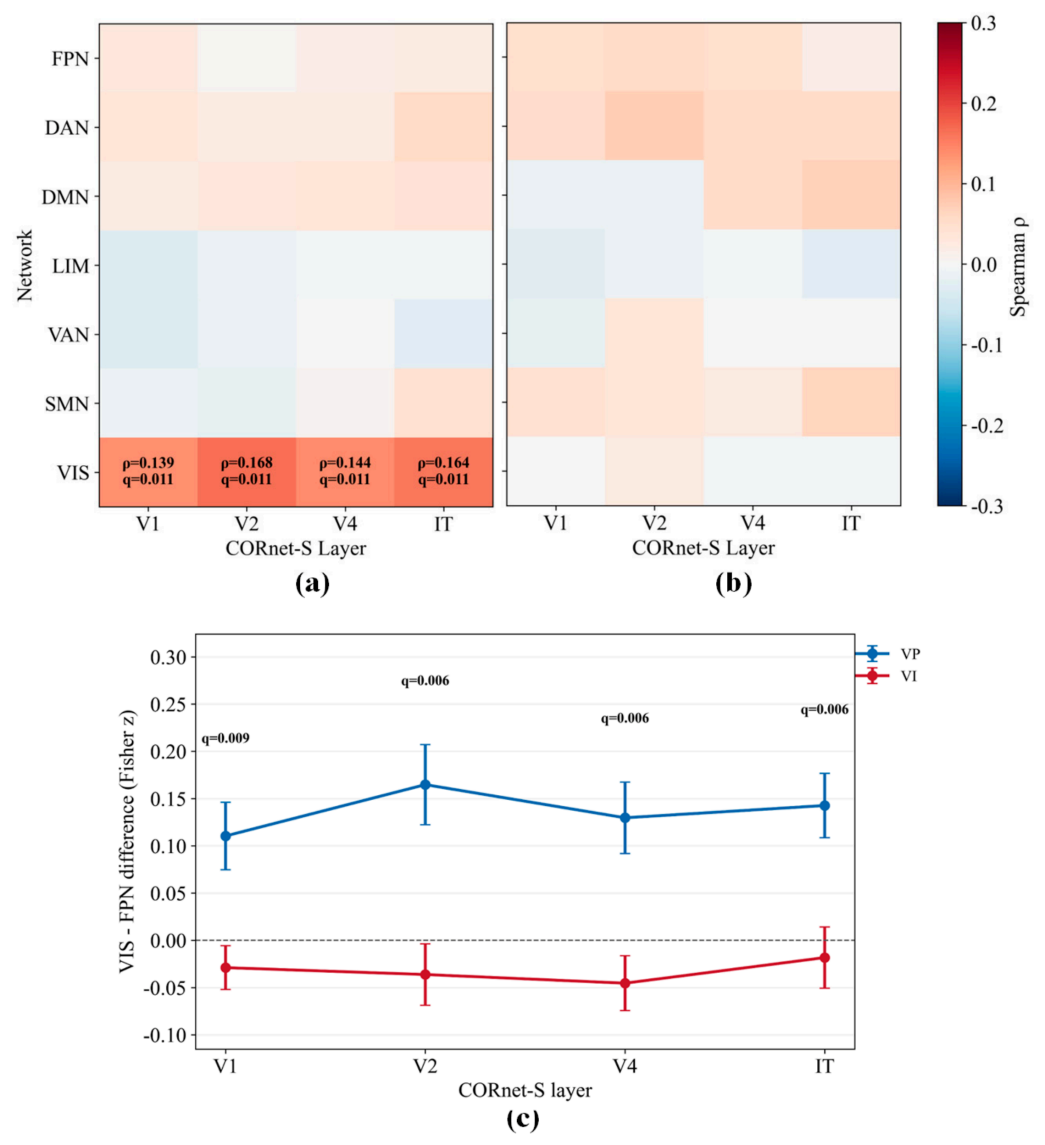
Representational similarity between CORnet-S hierarchical features and functional brain networks. (**a**) Results for the VP condition; (**b**) results for the VI condition; (**c**) task × network interaction analysis showing the within-task VIS–FPN representational contrasts for VP and VI across CORnet-S stages. In (**a**,**b**), the *y*-axis represents functional brain networks, and the *x*-axis represents CORnet-S hierarchical stages; values indicate Spearman correlation coefficients. In (**c**), the *y*-axis represents the VIS–FPN difference in Fisher *z*-transformed RSA coefficients, and the vertical separation between the VP and VI curves corresponds to the task × network interaction. Error bars represent the standard error of the mean (SEM) across participants. *q* indicates the FDR-adjusted *p*-value.

**Figure 11:**
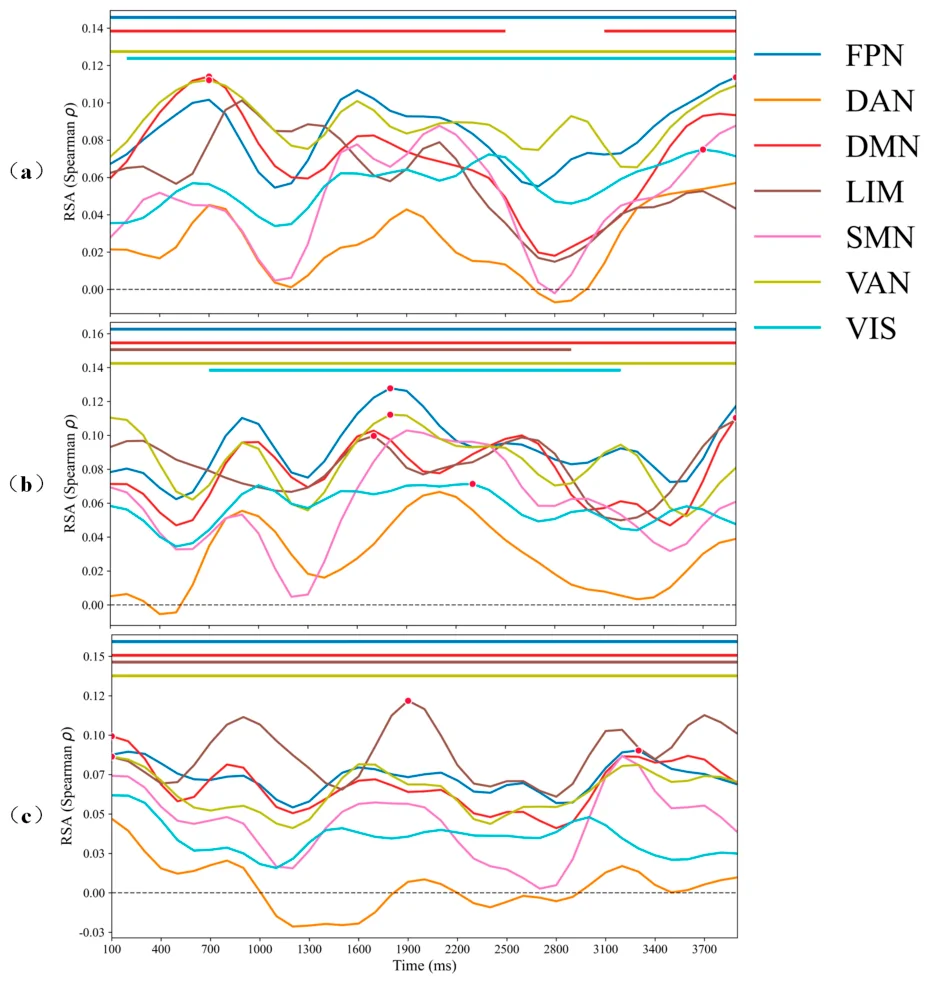
Time-resolved EEG–fMRI representational similarity analysis across different frequency bands. The *y*-axis represents Spearman correlation coefficients (*ρ*), and the *x*-axis represents time (ms), with time zero corresponding to the onset of the imagery period. Different colored curves represent different functional brain networks. Horizontal solid lines above the curves indicate significant temporal clusters after cluster-based permutation correction. Red dots on the curves indicate peak correlation points, defined as the maximum correlation values within significant temporal clusters: (**a**) alpha band; (**b**) beta band; (**c**) gamma band.

**Figure 12:**
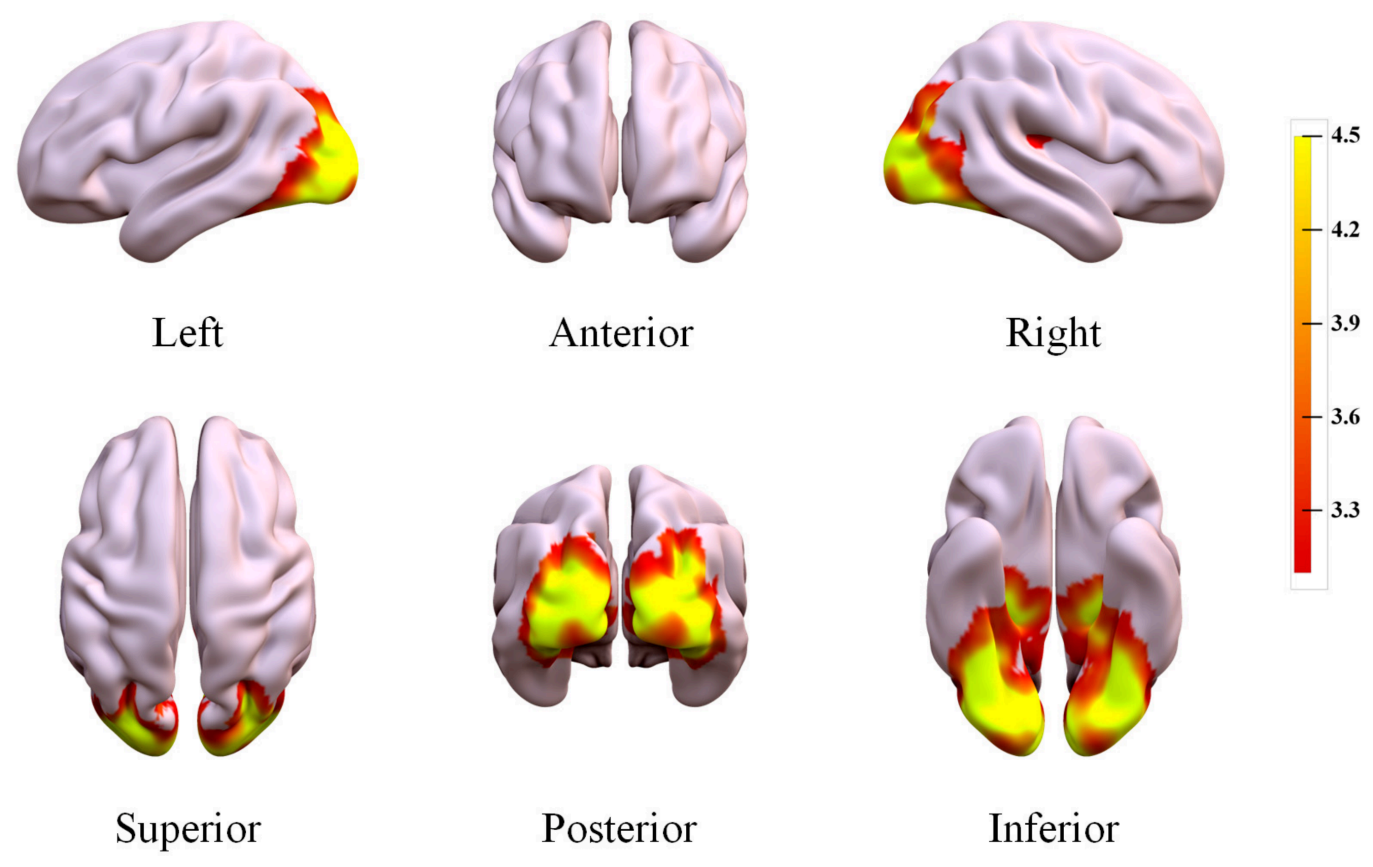
Group-level differential activation between VP and VI conditions (VP > VI).

**Figure 13:**
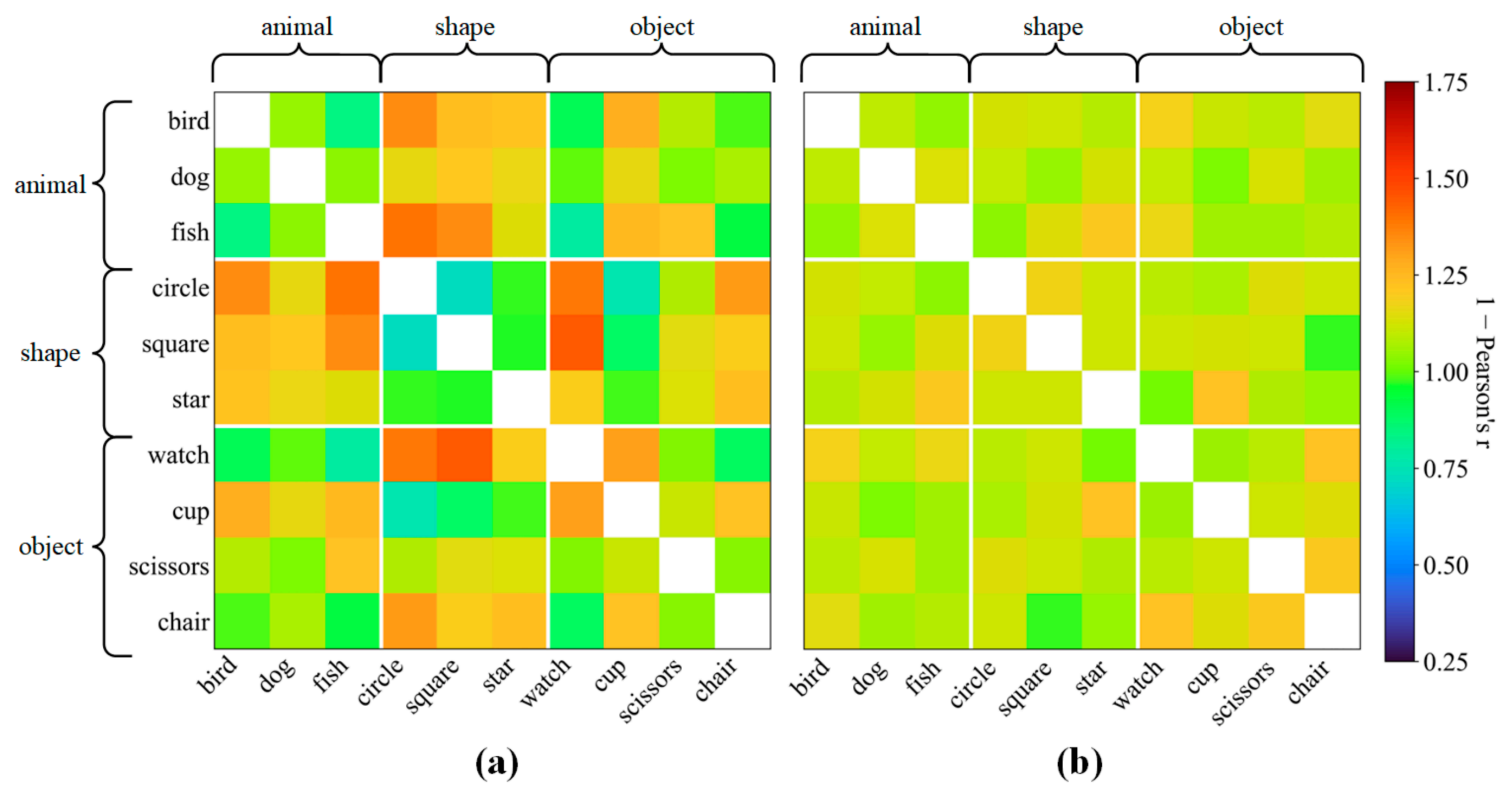
Representational dissimilarity matrices of the VIS network during VP and VI conditions. (**a**) Group-averaged RDM of the VIS network during the VP condition; (**b**) group-averaged RDM of the VIS network during the VI condition.

**Figure 14:**
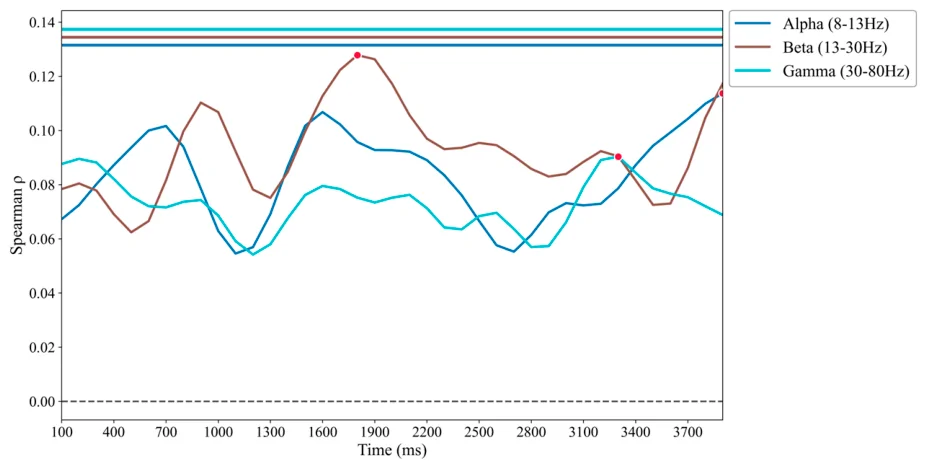
Representational similarity analysis results of the FPN across alpha, beta, and gamma frequency bands. Red dots on the curves indicate peak correlation points, defined as the maximum correlation values within significant temporal clusters.

**Table 1:** Whole-brain activation clusters during the VP task.

Cluster	Voxels	Peak MNI	Peak Brain Region	Peak *t*	*q*
1	18,068	(−32, −72, −12)	Left Fusiform Gyrus	12.326	<0.001
2	529	(−20, −28, −2)	Left Lateral Geniculate Nucleus	7.219	0.009
3	780	(22, −30, 0)	Right Thalamus	7.319	0.002
4	1718	(44, −4, 56)	Right Middle Frontal Gyrus	6.452	<0.001
5	949	(−44, −4, 56)	Left Precentral Gyrus	6.136	<0.001

**Table 2:** Whole-brain activation clusters during the VI task.

Cluster	Voxels	Peak MNI	Peak Brain Region	Peak *t*	*q*
1	801	(−54, 6, 18)	Left Inferior Frontal Gyrus, Opercular Part	5.151	0.018
2	653	(−38, −44, 38)	Left Inferior Parietal Lobule	4.101	0.018
3	680	(−2, 22, 46)	Left Supplementary Motor Area	4.176	0.018

**Table 3:** Whole-brain activation clusters for the VP > VI.

Cluster	Voxels	Peak MNI	Peak Brain Region	Peak *t*	*q*
1	20,980	(32, −64, −14)	Right Fusiform Gyrus	11.370	<0.001

## Data Availability

The datasets of the current study are available from the corresponding authors upon reasonable request due to restrictions related to participant privacy, informed consent, and ethical requirements.

## Supplementary Materials

The following supporting information can be downloaded at: https://www.mdpi.com/article/10.3390/brainsci16101024/s1, Table S1: RSA statistical results of CORnet-S for VP task within FPN; Table S2: RSA statistical results of CORnet-S for VP task within VIS; Table S3: RSA statistical results of CORnet-S for VI task within FPN; Table S4: RSA statistical results of CORnet-S for VI task within VIS; Table S5: statistical results of Task_x_Network_interaction; Table S6: RSA results of EEG-fMRI (8–13 Hz alpha band); Table S7: RSA results of EEG-fMRI (13–30 Hz beta band); Table S8: RSA results of EEG-fMRI (30–80 Hz Gamma band).
