# Supplementary material for: Network-Resolved and Frequency-Specific EEG–fMRI Representational Similarity Analysis of Visual Imagery
Source: Brain Sci. 2026 Sep 25;16(10):1024. doi: 10.3390/brainsci16101024 (PMC13702608; doi:10.3390/brainsci16101024)
Supplement: Supplementary file 1 [file brainsci-16-01024-s001.zip › brainsci-4545160-supplementary.pdf]

Supplementary Materials

Table S1 RSA statistical results of CORnet-S for VP task within FPN

V1	p_perm	p_fdr	p_fwe
V1	0.2735	0.8543	1.0000
V2	0.8884	0.8884	1.0000
V4	0.6144	0.8543	1.0000
IT	0.6407	0.8543	1.0000

Table S2 RSA statistical results of CORnet-S for VP task within VIS.

V1	p_perm	p_fdr	p_fwe
V1	0.0047,	0.0115	0.0188
V2	0.0060	0.0115	0.0260
V4	0.0115	0.0115	0.0460
IT	0.0103	0.0115	0.0412

Table S3 RSA statistical results of CORnet-S for VI task within FPN

V1	p_perm	p_fdr	p_fwe
V1	0.0848	0.2977	0.3392
V2	0.2641	0.2977	1.0000
V4	0.2977	0.2977	1.0000
IT	0.2016	0.2977	0.8063

Table S4 RSA statistical results of CORnet-S for VI task within VIS

V1	p_perm	p_fdr	p_fwe
V1	0.5390	0.7187	1.0000
V2	0.8128	0.8128	1.0000
V4	0.4101	0.7187	1.0000
IT	0.4977	0.7187	1.0000

Table S5 statistical results of Task_x_Network_interaction

layer	mean_diff_z	t-value	p-value	p-value ((BH-FDR correct)	p-value (Bonferroni correct)
V1	0.1394	3.419	0.009033	0.009033	0.036133
V2	0.201	3.411	0.004639	0.006185	0.018555
V4	0.1751	3.904	0.002441	0.006185	0.009766
IT	0.161	3.263	0.004395	0.006185	0.017578

Table S6 RSA results of EEG-fMRI (8–13 Hz alpha band)

network	cluster_id	start_ms	end_ms	network_p(BH-FDR correct)	network_p(Bonferroni correct)
FPN	1	100	3900	0.0007	0.0007
DAN	1	3300	3900	0.1250	0.8749
DMN	1	100	2500	0.0156	0.0518
DMN	2	3100	3900	0.0156	0.0518
LIM	1	200	400	0.0990	0.4949
LIM	2	600	1600	0.0990	0.4949
LIM	3	2000	2200	0.0990	0.4949
SMN	1	1500	2300	0.1062	0.6369
SMN	2	3500	3900	0.1062	0.6369
VAN	1	100	3900	0.0007	0.0014
VIS	1	200	3900	0.0156	0.0623

Table S7 RSA results of EEG-fMRI (13–30 Hz beta band)

network	cluster_id	start_ms	end_ms	network_p(BH-FDR correct)	network_p(Bonferroni correct)
FPN	1	100	3900	0.0038	0.0056
DAN	1	800	1000	0.1368	0.9575
DAN	2	1800	2300	0.1368	0.9575
DAN	3	3700	3900	0.1368	0.9575
DMN	1	100	3900	0.0047	0.0140
LIM	1	100	2900	0.0400	0.2002
LIM	2	3500	3900	0.0400	0.2002
SMN	1	100	300	0.0503	0.3017
SMN	2	1500	3100	0.0503	0.3017
SMN	3	3700	3900	0.0503	0.3017
VAN	1	100	3900	0.0038	0.0077
VIS	1	100	500	0.0212	0.0847
VIS	2	700	3200	0.0212	0.0847
VIS	3	3400	3900	0.0212	0.0847

Table S8 RSA results of EEG-fMRI (30–80 Hz Gamma band)

network	cluster_id	start_ms	end_ms	network_p(BH-FDR correct)	network_p(Bonferroni correct)
FPN	1	100	3900	0.0110	0.0329
DAN	None	None	None	1.0000	1.0000
DMN	1	100	3900	0.0077	0.0077
LIM	1	100	3900	0.0448	0.1792
SMN	1	100	400	0.1864	1.0000
SMN	2	1600	2000	0.1864	1.0000
SMN	3	3100	3400	0.1864	1.0000
VAN	1	100	3900	0.0110	0.0280
VIS	1	100	400	0.1289	0.6446
VIS	2	1400	2300	0.1289	0.6446
VIS	3	2800	3100	0.1289	0.6446
